# Investigation of mixing viscoplastic fluid with a modified anchor impeller inside a cylindrical stirred vessel using Casson–Papanastasiou model

**DOI:** 10.1038/s41598-022-22415-6

**Published:** 2022-10-20

**Authors:** Kada Benhanifia, Fares Redouane, Rahmani Lakhdar, Mebarki Brahim, Khaled Al-Farhany, Wasim Jamshed, Mohamed R. Eid, Sayed M. El Din, Zehba Raizah

**Affiliations:** 1 Laboratory of Energy in Arid Region (ENERGARID), Faculty of Science and Technology, University of Tahri Mohamed Bechar, P.O. Box 417, 08000 Béchar, Algeria; 2 LGIDD, Department of Physics, Faculty of Science and Technology, University of Relizane, 48000 Relizane, Algeria; 3 Department of Mechanical Engineering, University of Al-Qadisiyah, Al-Qadisiyah, 58001 Iraq; 4 Department of Mathematics, Capital University of Science and Technology (CUST), Islamabad, 44000 Pakistan; 5 Department of Mathematics, Faculty of Science, New Valley University, Al-Kharga, 72511 Al-Wadi Al-Gadid Egypt; 6 Department of Mathematics, Faculty of Science, Northern Border University, Arar, 1321 Saudi Arabia; 7 Center of Research, Faculty of Engineering, Future University in Egypt, New Cairo, 11835 Egypt; 8 Department of Mathematics, College of Science, King Khalid University, Abha, Saudi Arabia

**Keywords:** Mathematics and computing, Physics

## Abstract

In process engineering as chemical and biotechnological industry, agitated vessels are commonly used for various applications; mechanical agitation and mixing are performed to enhance heat transfer and improve specific Physico-chemical characteristics inside a heated tank. The research subject of this work is a numerical investigation of the thermo-hydrodynamic behavior of viscoplastic fluid (Casson–Papanastasiou model) in a stirred tank, with introducing a new anchor impeller design by conducting some modifications to the standard anchor impeller shape. Four geometry cases have been presented for achieving the mixing process inside the stirred vessel, CAI; classical anchor impeller, AI1; anchor impeller with added horizontal arm blade, AI2 and AI3 anchor impeller with two and three added arm blades, respectively. The investigation is focused on the effect of inertia and plasticity on the thermo-hydrodynamic behavior (flow pattern, power consumption, and heat transfer) by varying the Reynolds number (Re = 1, 10, 100, 200), Bingham number (Bn = 1, 10, 50), in addition to the effect of geometry design in the overall stirred system parameters. The findings revealed an excellent enhancement of flow pattern and heat transfer in the stirred system relatively to the increase of inertia values. Also, an energy reduction has been remarked and the effect of anchor impeller shape. AI3 geometry design significantly improves the flow pattern and enhances heat transfer by an increased rate of 10.46% over the other cases.

## Introduction

Mechanical agitation and mixing are critical processes in various sectors, including chemical-pharmaceutical, petroleum metallurgy, etc. It was utilized in various processes, including polymerization, dispersing, emulsifying, suspending, and mass and heat transfer enhancement. The purpose of mixing is to obtain a specific degree of homogeneity within the stirred system. Temperature is a significant element in the majority of chemical reactions.

It significantly influences thermodynamics, kinetics, homogeneity process, and product quality. The thermal exchange may be accomplished by jacket heating or through the use of heat exchange internals in a stirred tank. In many industrial processes, the mixing of viscoplastic fluids is commonly carried out in stirred tanks, such as fermentation, pharmaceuticals, polymerization, personal and home care products, and food products. The study of thermal behavior inside the stirred tank is essential since they are essential in many relevant processes. Such as, they are critical to achieving the aimed reaction products, preventing the thermal loss control of reactions, and generating the appropriate super-saturation for the development of suitable crystals.

Computational Fluid Dynamics (CFD) is an effective software for studying complex fluid flows involving multi-physical fields. There are many numerical thermal behavioral studies and fluid flow in stirred tanks. Given the importance of mixing in various industrial processes, numerous studies consisted of experimental design and computational studies to develop the effectiveness of hydro-dynamic compositions and the operating constraints. Several studies were reported in the mechanical agitation literature by many authors; Bertrand et al.^1^, Rahmani et al.^2^, Ameur et al.^3–5^, Kamla^6,7^, Driss et al.^8^, Mokhefi^9^, and Jaszczur et al.^10^.

Rajasekaran et al.^11^ used ANSYS Fluent software to investigate milk's flow pattern and thermal behavior during heating inside a stirred vessel. They discovered that the heat transfer coefficient values simulated by a CFD program correspond to experimental data that are very congruent. Daza et al.^12^ numerical investigation of thermal behavior inside jacketed stirred vessel stirred. They acquire a correlation of dimensionless Nusselt number of stirred systems with an introducing six-blade turbine. Hami et al.^13^ numerically studied the hydro-thermal behavior of Newtonian fluid inside a stirred vessel by introducing inclined blades anchor. They found that the average Nusselt number decreased with increasing the angle degree of the blades. Benmoussa et al.^14,15,16^ analyzed the effect of plasticity and inertia on the thermal and hydrodynamic behavior inside a stirred tank. They discovered that the thermal behavior efficiency is affected by the hydrodynamic parameter's variation (inertia and plasticity) inside the stirred vessel. An experimental study of the agitated helical coil heat exchanger evaluated its performance using an Al_2_O_3_-water nanofluid by Srinivas et al.^17^. The result indicated increasing the rotation speed and temperature value of fluid energy up to 10.65% energy rate. Jaszczur et al.^10^ analyzed the heat transfer along the jacketed cylindrical vessel. The findings established Nusselt number correlations with Reynolds number and found the thermal behavior's dependence on inertia parameters. Additionally, additional experimental experiments were conducted to investigate and improve the thermal behavior of stirred vessels; SK et al.^18^, Perarasu et al.^19,20^, Kong et al.^21^, Naik and Vinod^22^, Mahir et al.^23^, Komoda and Date^24^.

However, it is crucial to note that few works include the viscoplastic fluid thermal studies in stirred tanks. Furthermore, no research implores this particular fluid (Casson–Papanastasiou) in the investigation process. Previous research indicates a shortage of mixed convection investigations in laminar streams induced by activists that generate predominantly tangential movements.

The empirical study of this research entails a thermal hydrodynamical examination of a Casson–Papanastasiou fluid and the effect of inertia ($Re=1{-}100$), plasticity ($Bn=1{-}100$), and the geometrical design of anchor impellers. Four different cases are under study; CAI: classical anchor impeller; AI1: anchor impeller with an added arm blade; AI2: anchor impeller with two added blades; AI3: anchor impeller with three added blades on the flow pattern, heat transfer intensity, and power consumption.

## Physical description

**Figure 1:**
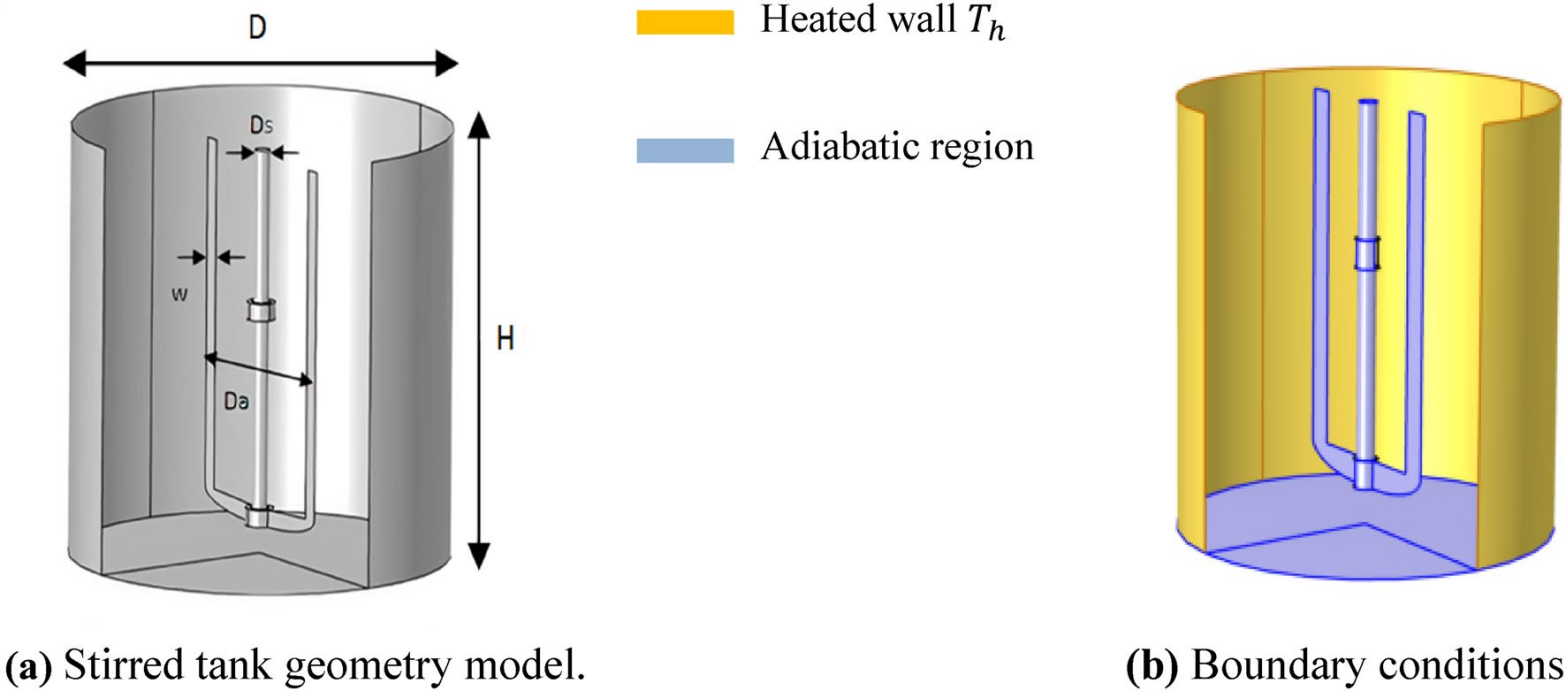
Geometry model of stirred tank and boundary conditions.

**Figure 2:**
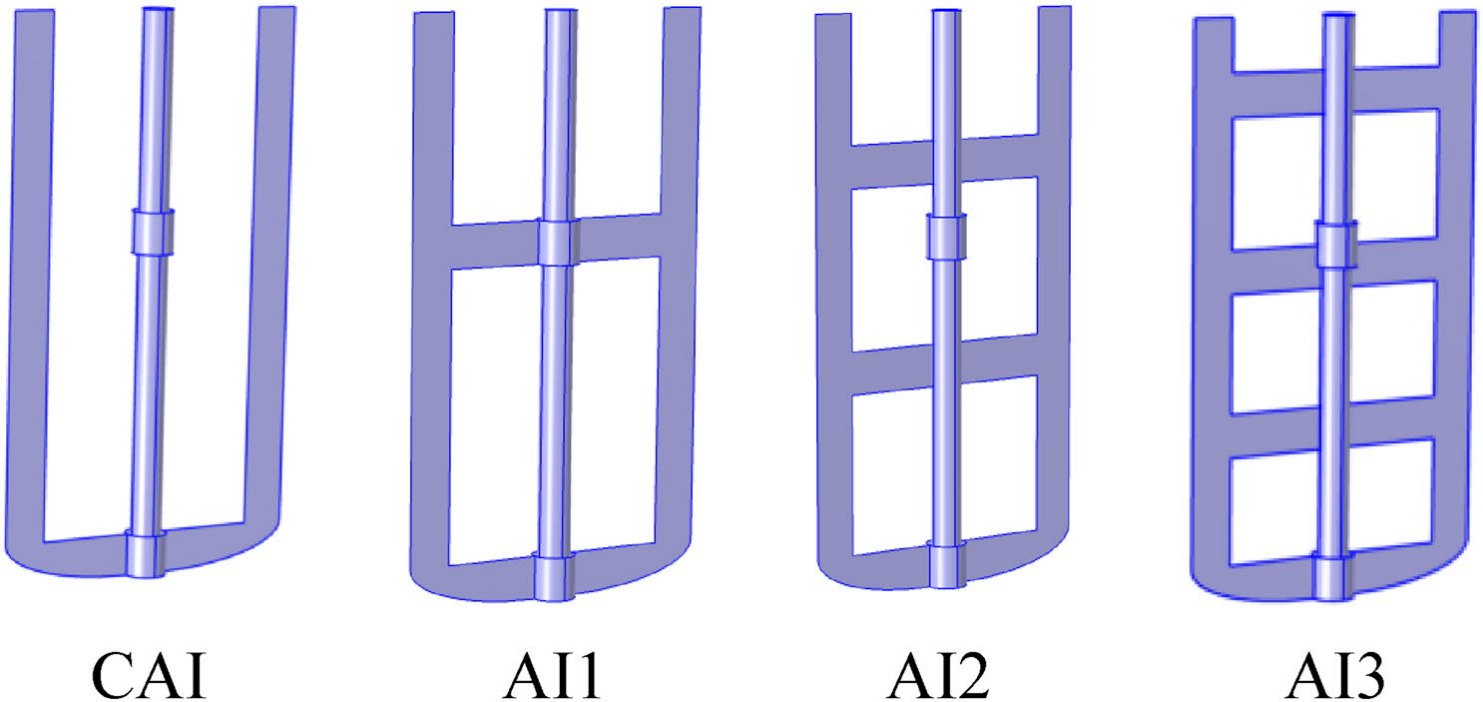
Different geometry configurations present in the stirred system.

Figure 1 illustrates the agitated vessel equipped with a classical anchor impeller. The stirred procedure contains a cylindric tank with a flatness bottom. The mixing process was investigated under a hot temperature ${T}_{h}$ at the tank's sidewall, while the anchor and the tank's bottom wall are assumed to be adiabatic. However, this agitated vessel was equipped with a different geometrical configuration of anchor impeller, as shown in Fig. 2. The first case is a Classical Anchor Impeller (CAI), the second case is Anchor Impeller with one added arm blade (AI1), the third case is an anchor impeller with two blades (AI2), and the fourth case is an anchor impeller with three blades (AI3). All geometry parameter details are described in Table 1.

**Table 1:** Geometry details of a stirred vessel system.

$D$	$H$	$d$	$w$	$ds$	$c$
0.3	0.3	0.15	0.02	0.02	0.03

## Mathematical model

**Figure 3:**
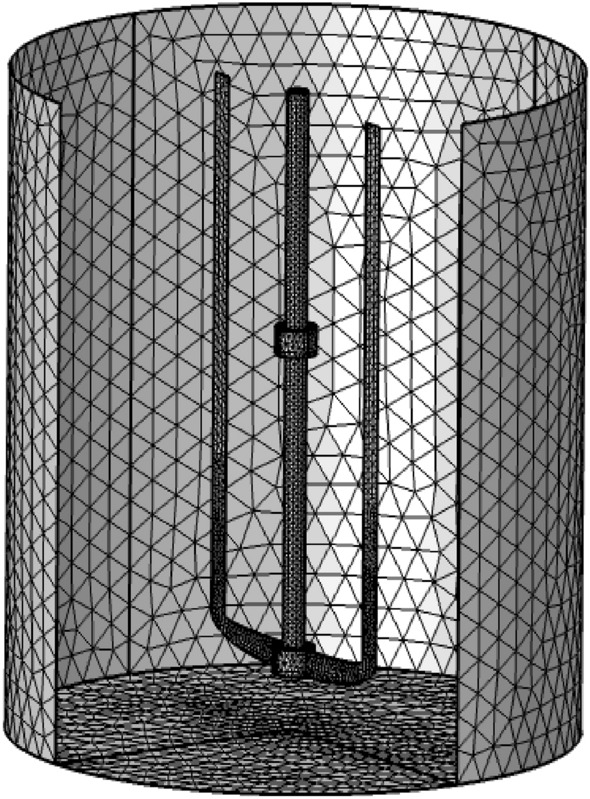
Unstructured mesh.

3-D numerical simulation of thermal and laminar mixing of viscoplastic fluid inside the stirred vessel. This investigation was performed with the CFD Code that solved the momentum and energy equations based on the finite element methods using Galerkin's discretization with an unstructured mesh, as shown in Fig. 3. In the discretization of the computational domain, the tetrahedral mesh was introduced, and it is particularly suitable for representing the geometrical domain due to its high adaptability to curved surfaces.

### Mesh test

We opted for a finer mesh for the 3D study, for which the results mentioned in Table 2 remain unchanged. In addition, the computation time is more important. It should be noted that, that the convergence criterion relates to the values ​​of each of the dimensionless dependent variables whose error must be lower than 10^–6^. The mesh test is done under the condition that ($Re=100$, $\mu =0.01$ Pa s, and $\tau =1\; \text{Pa}$).

**Table 2:** Mesh independence test.

Mesh type	Mesh element	$Nu$	CPU time
Normal	288,258	0.6907	3 h 32 min
Fine	1,024,653	0.70715	8 h 19 min
Finer	2,875,942	0.70721	15 h 52 min
Extra mesh	3,600,253	0.70724	21 h 11 min

### Problem formulation

#### Dimensional equation

##### Continuity equation

1 $$\frac{\partial U}{\partial X}+\frac{\partial V}{\partial Y}+\frac{dW}{dZ}=0,$$

##### Momentum equation

2 $$\rho \left(\frac{\partial U}{\partial t}+U\frac{\partial U}{\partial X}+V\frac{\partial U}{\partial Y}+W\frac{\partial U}{\partial Z}\right)=-\frac{\partial P}{\partial X}+\left(1+\frac{1}{\beta }\right)\left[\frac{\partial }{\partial X}\left(2\mu \frac{\partial U}{\partial X}\right)+\frac{\partial }{\partial Y}\left(\mu \frac{\partial U}{\partial Y}+\frac{\partial V}{\partial X}\right)+\frac{\partial }{\partial z}\left(\mu \frac{\partial U}{\partial Z}+\frac{\partial W}{\partial X}\right)\right],$$

3 $$\rho \left(\frac{\partial V}{\partial t}+U\frac{\partial V}{\partial X}+V\frac{\partial V}{\partial Y}+W\frac{\partial V}{\partial Z}\right)=-\frac{\partial P}{\partial Y}+\left(1+\frac{1}{\beta }\right)\left[\frac{\partial }{\partial X}\left(\mu \frac{\partial U}{\partial Y}+\frac{\partial V}{\partial X}\right)+\frac{\partial }{\partial Y}\left(2\mu \frac{\partial V}{\partial Y}\right)+\frac{\partial }{\partial z}\left(\mu \frac{\partial U}{\partial Z}+\frac{\partial W}{\partial X}\right)\right],$$

4 $$\rho \left(\frac{\partial W}{\partial t}+U\frac{\partial W}{\partial X}+V\frac{\partial W}{\partial Y}+W\frac{\partial W}{\partial Z}\right)=-\frac{\partial P}{\partial Z}+\left(1+\frac{1}{\beta }\right)\left[\frac{\partial }{\partial X}\left(\mu \frac{\partial U}{\partial Y}+\frac{\partial W}{\partial X}\right)+\frac{\partial }{\partial Y}\left(\mu \frac{\partial V}{\partial Z}+\frac{\partial W}{\partial Y}\right)+\frac{\partial }{\partial Z}\left(2\mu \frac{\partial W}{\partial Z}\right)\right],$$

##### Energy equation

5 $$\frac{\partial T}{\partial t}+U\frac{\partial T}{\partial X}+V\frac{\partial T}{\partial Y}+W\frac{\partial T}{\partial Z}=\alpha \left(\frac{{\partial }^{2}T}{\partial {X}^{2}}\right)+\left(\frac{{\partial }^{2}T}{\partial {Y}^{2}}\right)+\left(\frac{{\partial }^{2}T}{\partial {Z}^{2}}\right).$$

##### Working fluid in the dimensional equation

6 $$\mu ={\mu }_{p}+\sqrt{\frac{{\tau }_{0}}{\gamma }}\left(1-\mathit{exp}\left(\sqrt{-m\stackrel{.}{\gamma }}\right)\right).$$

To model the stress-deformation behavior of yield stress fluids, the Casson–Papanastasiou constitutive equation has been modified by Papanastasiou^25^.

Here ${\mu }_{p}$ represents the plastic viscosity, $\text{m}$ is the stress growth exponent called a regularization parameter, $\dot{\gamma }$ is the shear rate, and $\tau$ is the shear stress.

##### Dimensionless parameters

The below dimensionless parameters were used to reduce the number of variables and also to convert the dimensional governing equations to their dimensionless form:

7 $$\left.\begin{array}{l}{X}^{*}=\frac{2X}{D},{Y}^{*}=\frac{2Y}{D},{Z}^{*}=\frac{2Z}{D},{U}^{*}=\frac{U}{\pi ND},{V}^{*}=\frac{V}{\pi ND},{W}^{*}=\frac{W}{\pi ND},\\ {P}^{*}=\frac{P}{\rho {\left(\pi ND\right)}^{2}},{\dot{\gamma }}^{*}=\frac{2}{\pi N{D}^{2}}\gamma ,{T}^{*}=\frac{T-{T}_{C}}{{T}_{h}-{T}_{C}},{t}^{*}=2\pi Nt.\end{array}\right\}$$

#### Dimensionless equation

##### Continuity equation

8 $$\frac{\partial {U}^{*}}{\partial X}+\frac{\partial {V}^{*}}{\partial Y}+\frac{d{W}^{*}}{dZ}=0,$$

##### Momentum equation

9 $$\left(\frac{\partial {U}^{*}}{\partial {t}^{*}}+{U}^{*}\frac{\partial {U}^{*}}{\partial {X}^{*}}+{V}^{*}\frac{\partial {U}^{*}}{\partial {Y}^{*}}+{W}^{*}\frac{\partial {U}^{*}}{\partial {Z}^{*}}\right)=-\frac{\partial {P}^{*}}{\partial {X}^{*}}+{\left(\frac{d}{D}\right)}^{2}\frac{1}{Re}\left(1+\frac{1}{\beta }\right)\left[\frac{\partial }{\partial {X}^{*}}\left(2{\mu }^{*}\frac{\partial {U}^{*}}{\partial {X}^{*}}\right)+\frac{\partial }{\partial {Y}^{*}}\left({\mu }^{*}\left(\frac{\partial {U}^{*}}{\partial {Y}^{*}}+\frac{\partial {V}^{*}}{\partial {X}^{*}}\right)\right)+\frac{\partial }{\partial {Z}^{*}}\left({\mu }^{*}\left(\frac{\partial {U}^{*}}{\partial {Z}^{*}}+\frac{\partial {W}^{*}}{\partial {X}^{*}}\right)\right)\right],$$

10 $$\left(\frac{\partial {V}^{*}}{\partial {t}^{*}}+{U}^{*}\frac{\partial {V}^{*}}{\partial {X}^{*}}+{V}^{*}\frac{\partial {V}^{*}}{\partial {Y}^{*}}+{W}^{*}\frac{\partial {V}^{*}}{\partial {Z}^{*}}\right)=-\frac{\partial {P}^{*}}{\partial {Y}^{*}}+{\left(\frac{d}{D}\right)}^{2}\frac{1}{Re}\left(1+\frac{1}{\beta }\right)\left[\frac{\partial }{\partial {X}^{*}}\left({\mu }^{*}\left(\frac{\partial {U}^{*}}{\partial {Y}^{*}}+\frac{\partial {V}^{*}}{\partial {X}^{*}}\right)\right)+\frac{\partial }{\partial {Y}^{*}}\left(2{\mu }^{*}\frac{\partial {V}^{*}}{\partial {Y}^{*}}\right)+\frac{\partial }{\partial {Z}^{*}}\left({\mu }^{*}\left(\frac{\partial {U}^{*}}{\partial {Z}^{*}}+\frac{\partial {W}^{*}}{\partial {X}^{*}}\right)\right)\right],$$

11 $$\left(\frac{\partial {W}^{*}}{\partial {t}^{*}}+{U}^{*}\frac{\partial {W}^{*}}{\partial {X}^{*}}+{V}^{*}\frac{\partial {W}^{*}}{\partial {Y}^{*}}+{W}^{*}\frac{\partial {W}^{*}}{\partial {Z}^{*}}\right)-\frac{\partial {P}^{*}}{\partial {Y}^{*}}+{\left(\frac{d}{D}\right)}^{2}\frac{1}{Re}\left(1+\frac{1}{\beta }\right)\left[\frac{\partial }{\partial {X}^{*}}\left({\mu }^{*}\left(\frac{\partial {U}^{*}}{\partial {Y}^{*}}+\frac{\partial {V}^{*}}{\partial {X}^{*}}\right)\right)+\frac{\partial }{\partial {Y}^{*}}\left({\mu }^{*}\left(\frac{\partial {U}^{*}}{\partial {Z}^{*}}+\frac{\partial {W}^{*}}{\partial {X}^{*}}\right)\right)+\frac{\partial }{\partial {Z}^{*}}\left(2{\mu }^{*}\frac{\partial {W}^{*}}{\partial {Z}^{*}}\right)\right],$$

##### Energy equation

12 $$\frac{\partial {T}^{*}}{\partial {t}^{*}}+{U}^{*}\frac{\partial {T}^{*}}{\partial {X}^{*}}+{V}^{*}\frac{\partial {T}^{*}}{\partial {Y}^{*}}+{W}^{*}\frac{\partial {T}^{*}}{\partial {Z}^{*}}={\left(\frac{d}{D}\right)}^{2}\frac{1}{RePr}\left(\frac{{\partial }^{2}{T}^{*}}{\partial {{X}^{*}}^{2}}\right)+\left(\frac{{\partial }^{2}{T}^{*}}{\partial {{Y}^{*}}^{2}}\right)+\left(\frac{{\partial }^{2}{T}^{*}}{\partial {{Z}^{*}}^{2}}\right)$$

##### Dimensionless Casson–Papanastasiou fluid equation

13 $${\mu }^{*}=1+\sqrt{\frac{\text{Bn}}{{\dot{\gamma }}^{*}}}\left(1-\mathit{exp}\left(\sqrt{-M{\dot{\gamma }}^{*}}\right)\right),$$

##### Bingham numbers

14 $$Bn=\frac{\tau D}{{\mu }_{p}N},$$

##### Reynolds number

15 $$\mathit{Re}=\frac{\rho N{d}^{2}}{\mu },$$

##### Nusselt number

The average Nusselt is calculated by integrating local Nusselt $Nu$ along the hot wall as follows.

16 $$\overline{Nu }=\frac{1}{\pi D}\int \frac{\partial T}{\partial X}+\frac{\partial T}{\partial Y}+\frac{\partial T}{\partial Z},$$

For the heat transfer phenomena in the stirred tank, the boundary condition is assumed a dimensionless parameter however, ${T}_{h}$ (signifies the hot wall temperature and supposed that ${T}_{h}=1$) and ${T}_{c}$ (signifies the temperature of the cold wall and presumed that ${T}_{c}=0$). In addition the Nusselt number $Nu$ can be defined as the heat transfer rate along the mixing operation and it is expressed as denoted in Eq. (16).

##### Power number

17 $$Np=\frac{P}{\rho {N}^{3}{d}^{5}},$$

The power consumption $P$ calculate by the following equation

18 $$P={\int }_{A}2\pi N .\left(x{F}_{y}-y{F}_{x}\right) \cdot dA,$$

where $$N$$ is the rotational speed, $$A$$ surface around the impeller, and $$({F}_{x}$$, $${F}_{y})$$ represent the $$x$$ and $$y-$$ force direction.

## Validation

**Figure 4:**
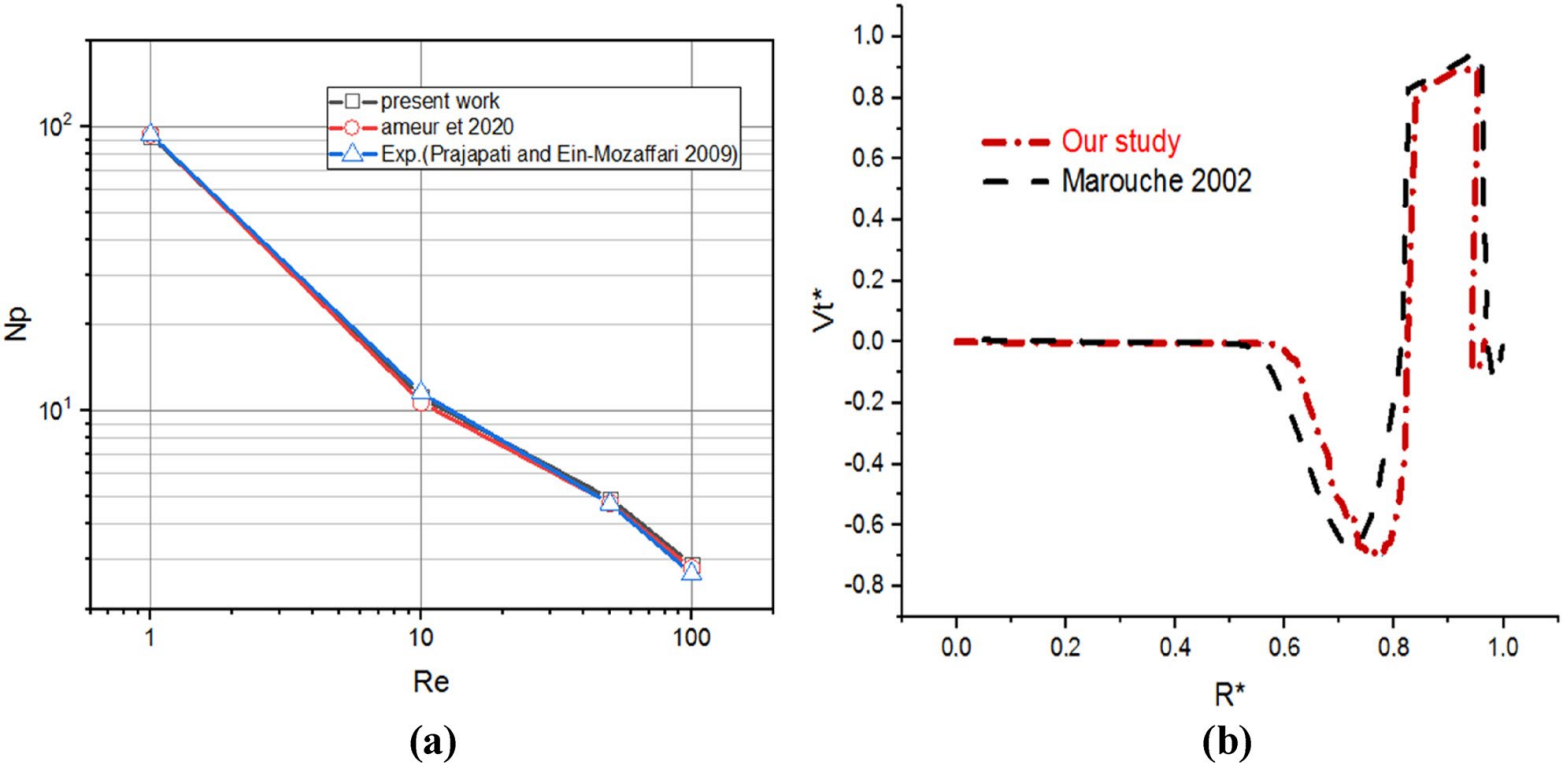
Validation curves for (**a**) power number and (**b**) tangential velocity.

To validate and verify the study of the computation code, numerical results were compared to those previously published in the literature to validate the numerical analysis used in Fig. 4. Power numbers and the tangential velocity obtained from the present study have been compared with the previous numerical and experimental studies existing in the mixing and mechanical agitation literature using anchor impellers, Ameur and Youcef^4^, Prajapati and Ein-Mozaffari^26^, and Marouche et al.^27^. Figure 4a illustrates the variation in power numbers as a function of Reynolds numbers ($Re=1, 10, 50,$ and $100$); when these works are compared to the results obtained in our investigation, a very excellent agreement is achieved. Marouche et al.^27^ referenced for comparison in our results in this research. The Bingham fluids were used with the same rheological and geometrical parameters (anchor impeller, a working fluid with the characterization $\mu =0.1$, $\tau =0.1$ with inertia value ($Re=13.8$). In Fig. 4b, the tangential velocity was of a high degree and slowly decreased as it approached the wall. Our findings are incredibly similar to the previous numerical results of Marouche et al.^27^, as illustrated in Fig. 4b.

## Results and discussion

### Inertia effect

The flow pattern is an essential criterion for previewing the mixing performance in the stirred tank system. The flow pattern is affected by the rotational speed of the anchor impeller and its design geometry. A different inertia value is tested ($Re=1, 10, 50,$ and $100$). Many anchor impeller shapes have been introduced to analyze their influence on the hydro-thermal behavior inside the mixing vessel system.

**Figure 5:**
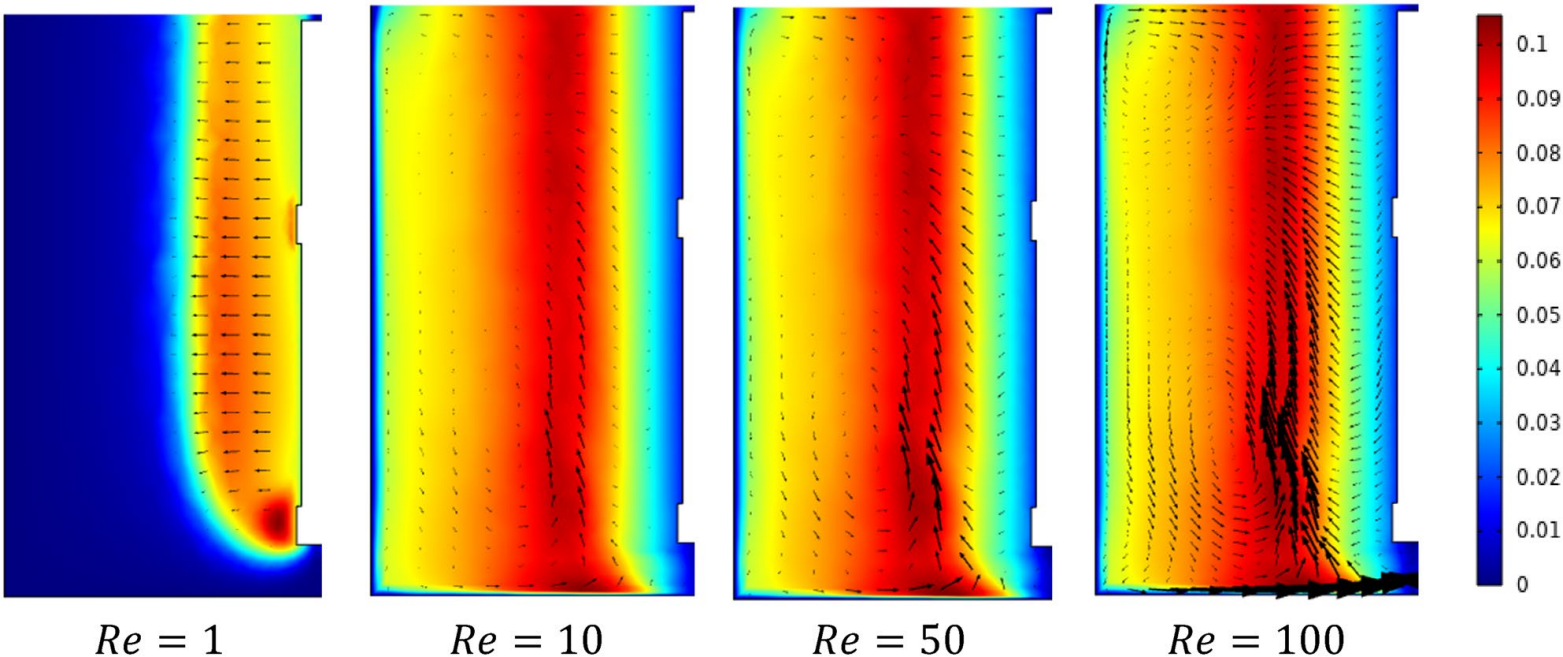
Contour velocity and arrow velocity field distribution in the vertical median section on the stirred tank for different Reynolds numbers.

**Figure 6:**
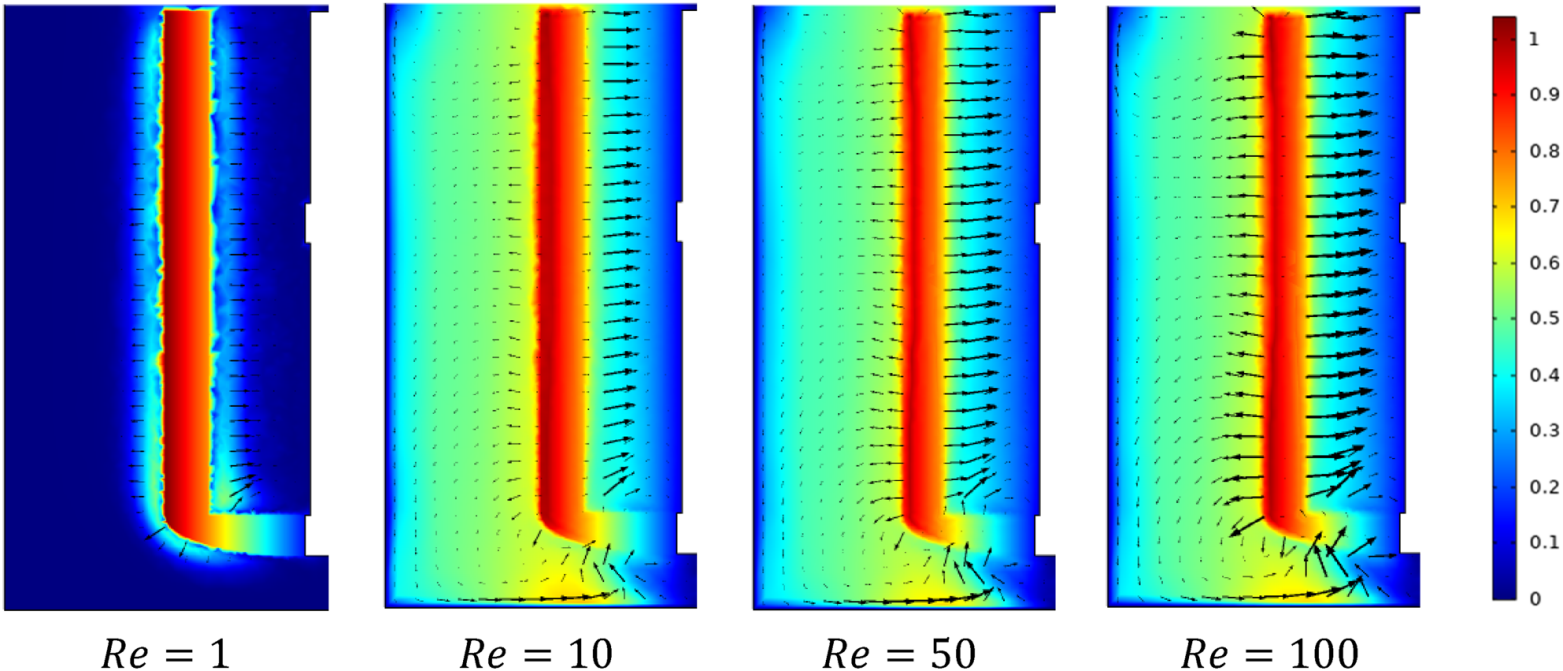
Contour velocity and arrow velocity filed distribution in the vertical impeller section on the stirred tank for different Reynolds numbers.

Figures 5 and 6 show the velocity magnitude and vectors filed velocity distribution along the vertical median plane and impeller plane, respectively, for different values of the Reynolds number. From the vector flow fields in the case of $Re=1$, it can remark a similarity of vector flow field flow along the vertical plane. It appears to parallel at all heights, explaining that the flow field is predominantly tangential. Furthermore, the limitation on the moving zone on the impeller area and low dimensionless velocity value, in this case, $V{t}^{*} = 1\times {10}^{-4}$.

With the increase of inertia value ($Re=10$ and $50$), an increase in the vectors field density around the impeller has been remarked, and a tiny change accompanied it on the flow pattern, an inclination of the vector field flow from the radial toward the axial direction and an expanding of moving zone toward the whole tank. In the same way, for the case $Re=100$, a high vector field density has been created near the impeller area compared to the previous cases. In this case, the flow pattern becomes greatly radial.

**Figure 7:**
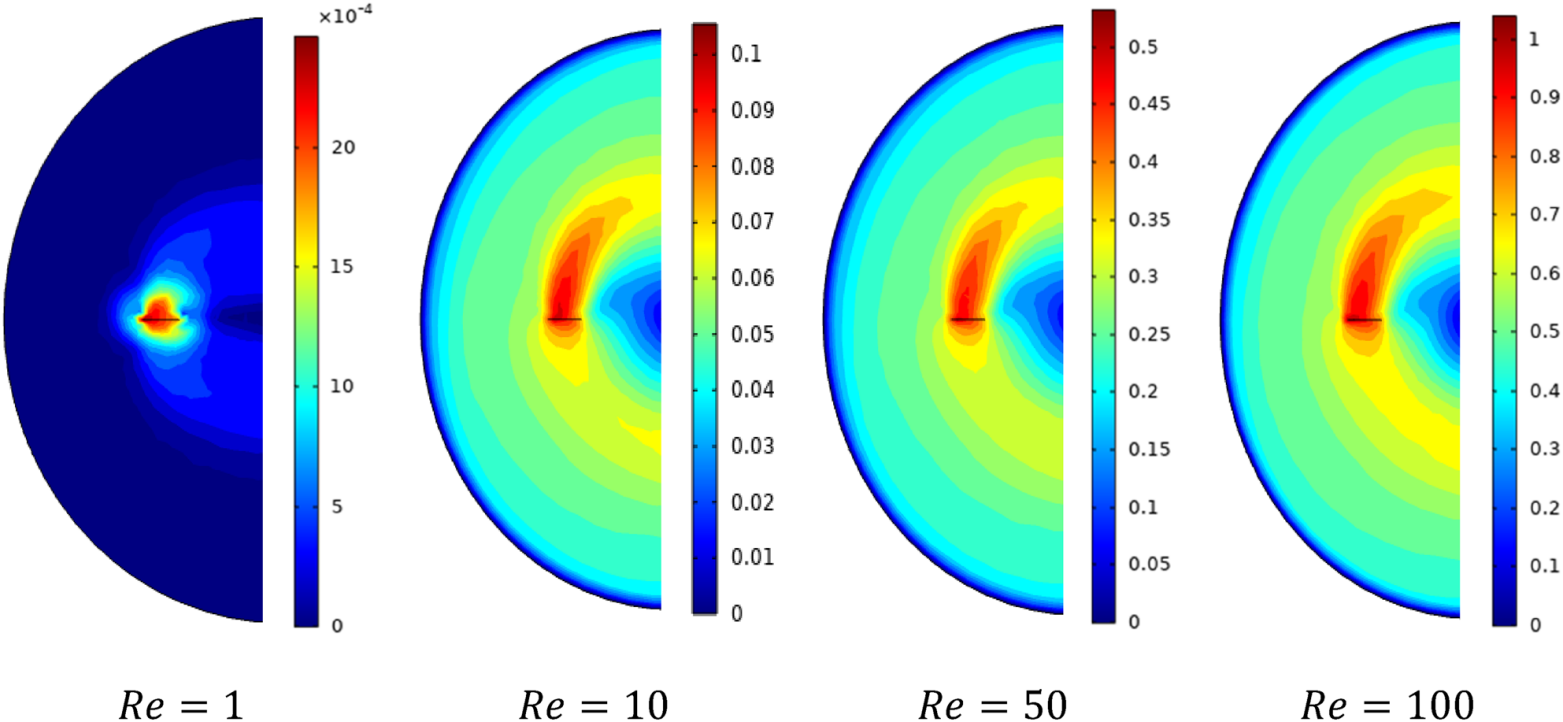
Contour velocity distribution in the horizontal impeller section on the stirred tank for different Reynolds numbers.

**Figure 8:**
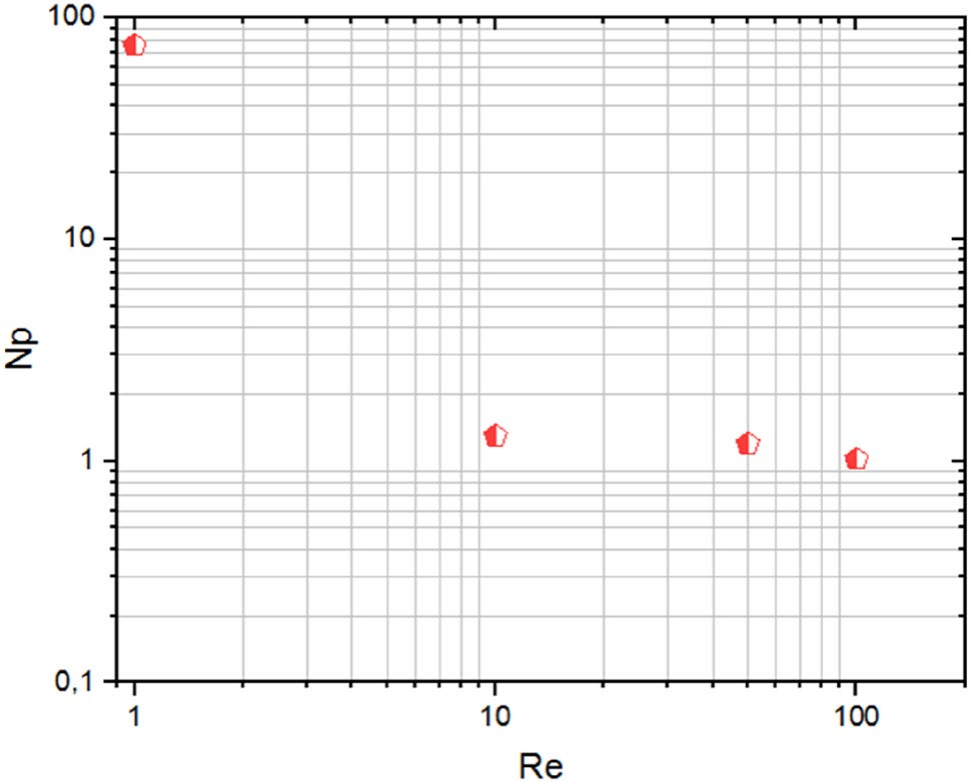
Power consumption versus different Reynolds numbers.

Figure 7 presents the contour velocity on the horizontal section of the vessel. A significant positive correlation between the inertia and moving well zone with the increase in inertia values and rise in velocity magnitude is recorded. That means an important variation in flow intensity along with stirred tank according to the rise of inertia value. Figure 8 shows the evolution of the power consumption ($Np$) as a function of the inertia value. The results indicate that the continuous increase of the Reynolds number reduces the energy required in the stirred system. The inertia parameter significantly influences energy consumed with the rise of this parameter, which means less power consumption is required. This rise of Reynolds minimizes the cost of energy consumed in this stirred vessel.

**Figure 9:**
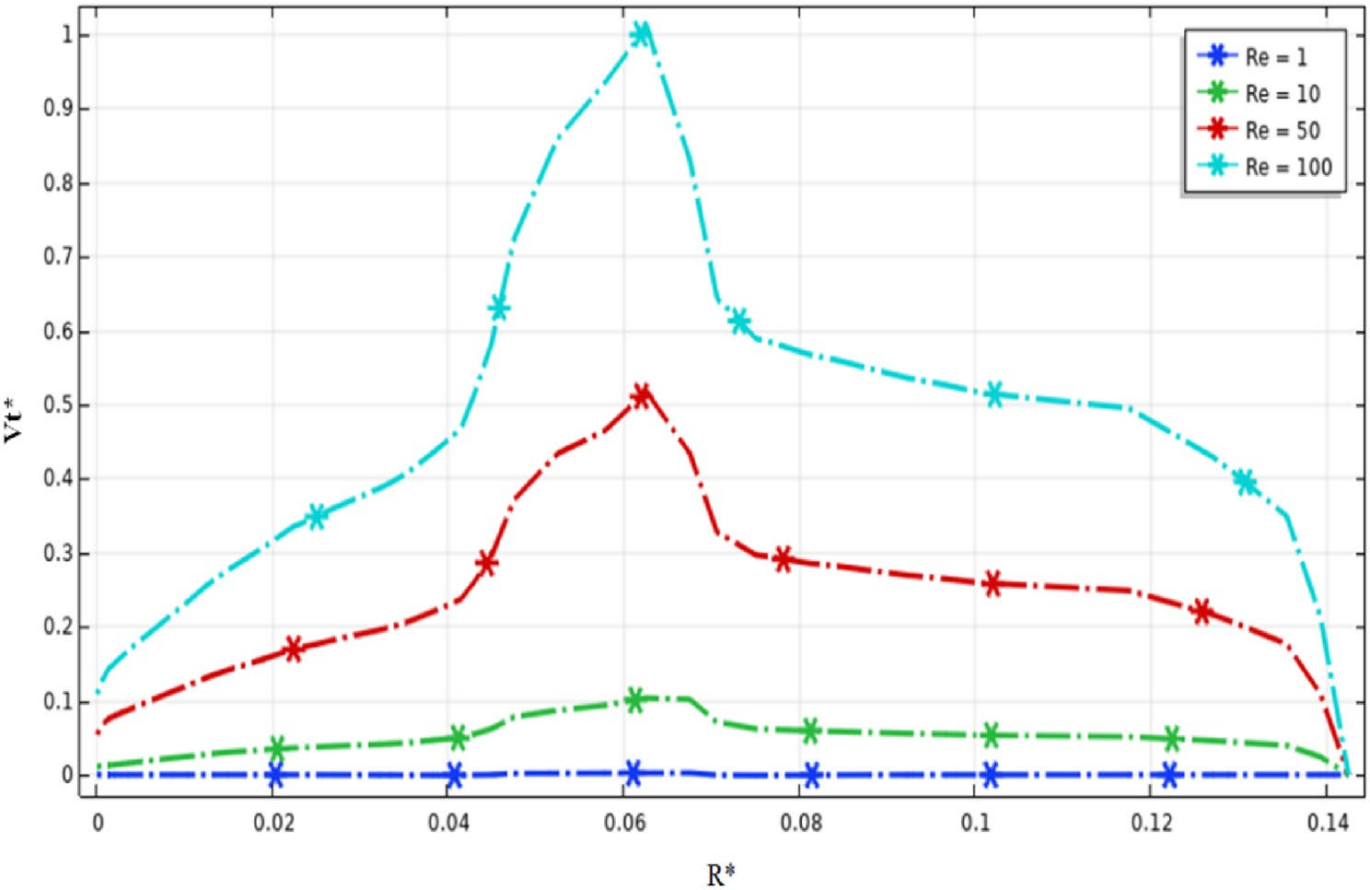
Velocity magnitude distribution $V{t}^{*}$ in the horizontal radial section on the stirred tank for different Reynolds numbers.

**Figure 10:**
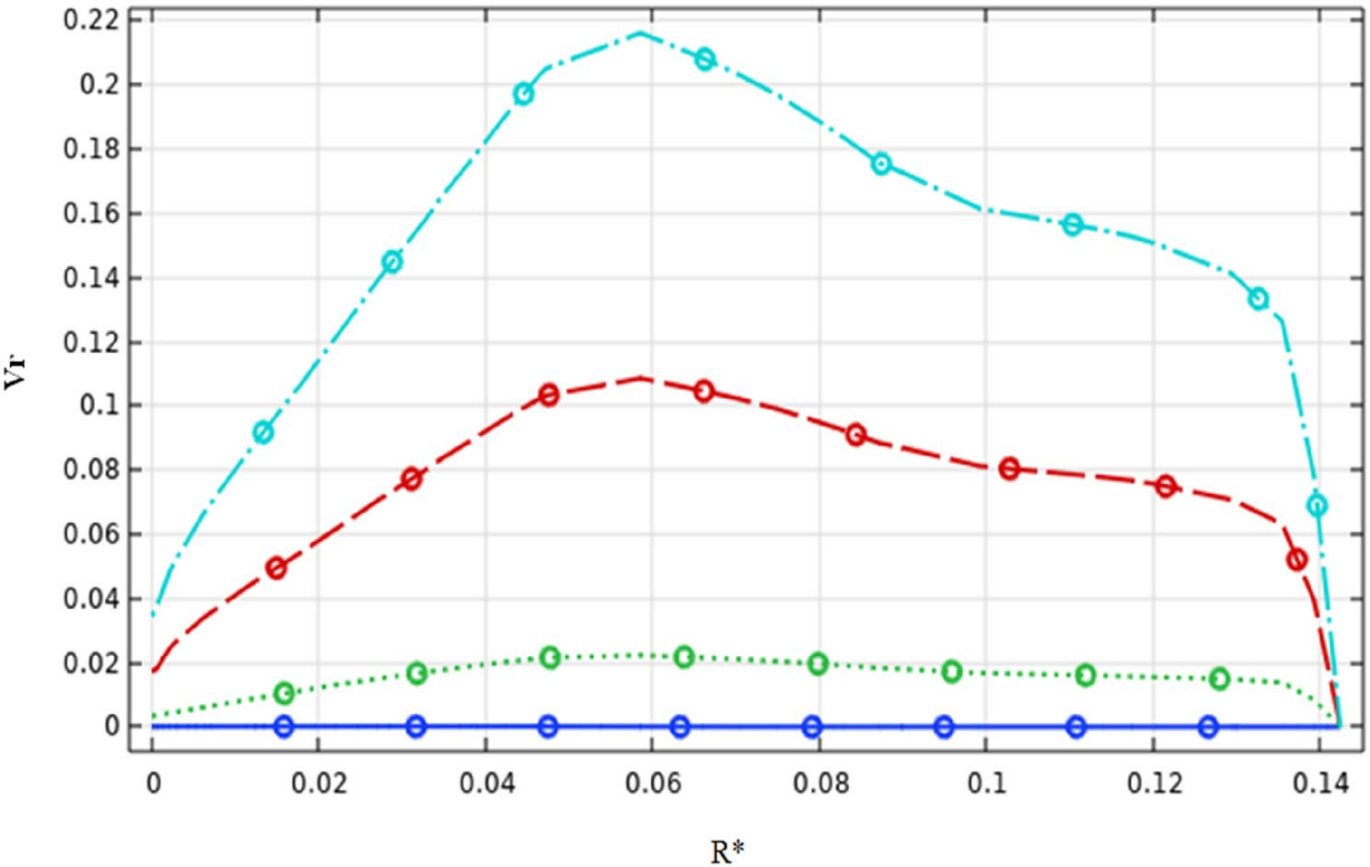
Velocity magnitude distribution $Vr$ in the horizontal median section on the stirred tank for different Reynolds numbers.

Figures 9 and 10 illustrate the velocity magnitude in the tangential direction (impeller plane) and the radial direction (median plane) along the vertical section of the stirred tank. Overall, it can be noted that the maximum velocity was almost located in the middle of the vessel strictly at the anchor region and it becomes decays at the immediate contact with the side vessel wall. The velocity distributions on the impeller plane are comparable to outcomes obtained by Kada et al.^28^ and Benmoussa^15^. Also, it can be observed that the maximum velocity on the median plane (radial velocity) becomes $V{r}^{*}=0.36$ and the maximum velocity value on the impeller plane (tangential velocity) is $V{t}^{*}=0.96$. As a result of this observation, we may conclude that tangential flow dominates in this stirred system; the same effect was found by Ameur^29^ and Mebarki et al.^30^.

**Figure 11:**
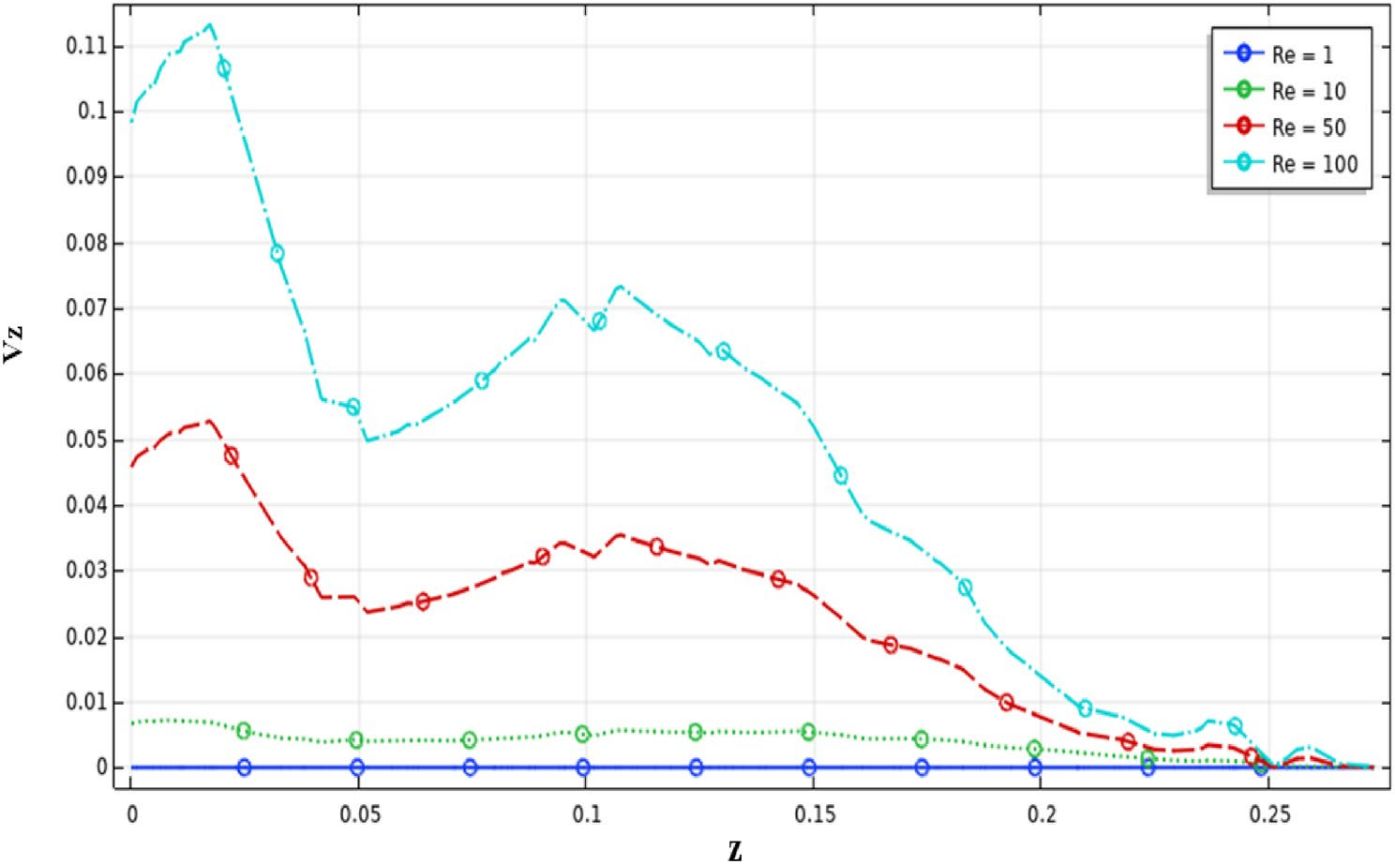
Velocity distribution in the vertical section $Vz$ on the stirred tank for different Reynolds numbers.

Analyzing the velocity distribution along the vertical axis precisely gives the flow structure inside the stirred tank system. Figure 11 illustrates the velocity distribution in the vertical axis of the vessel. It is apparent from this result that the maximum value of the $Vz$ is 0.11. Compared with the value obtained from the tangential velocity, we can note that $Vz$ is very small  compared to the tangential flow inside stirred tank. This means that they have a low impact on axial flow on the stirred tank, and the tangential flow usually is dominant in this system.

### Heat transfer on the stirred tank

**Figure 12:**
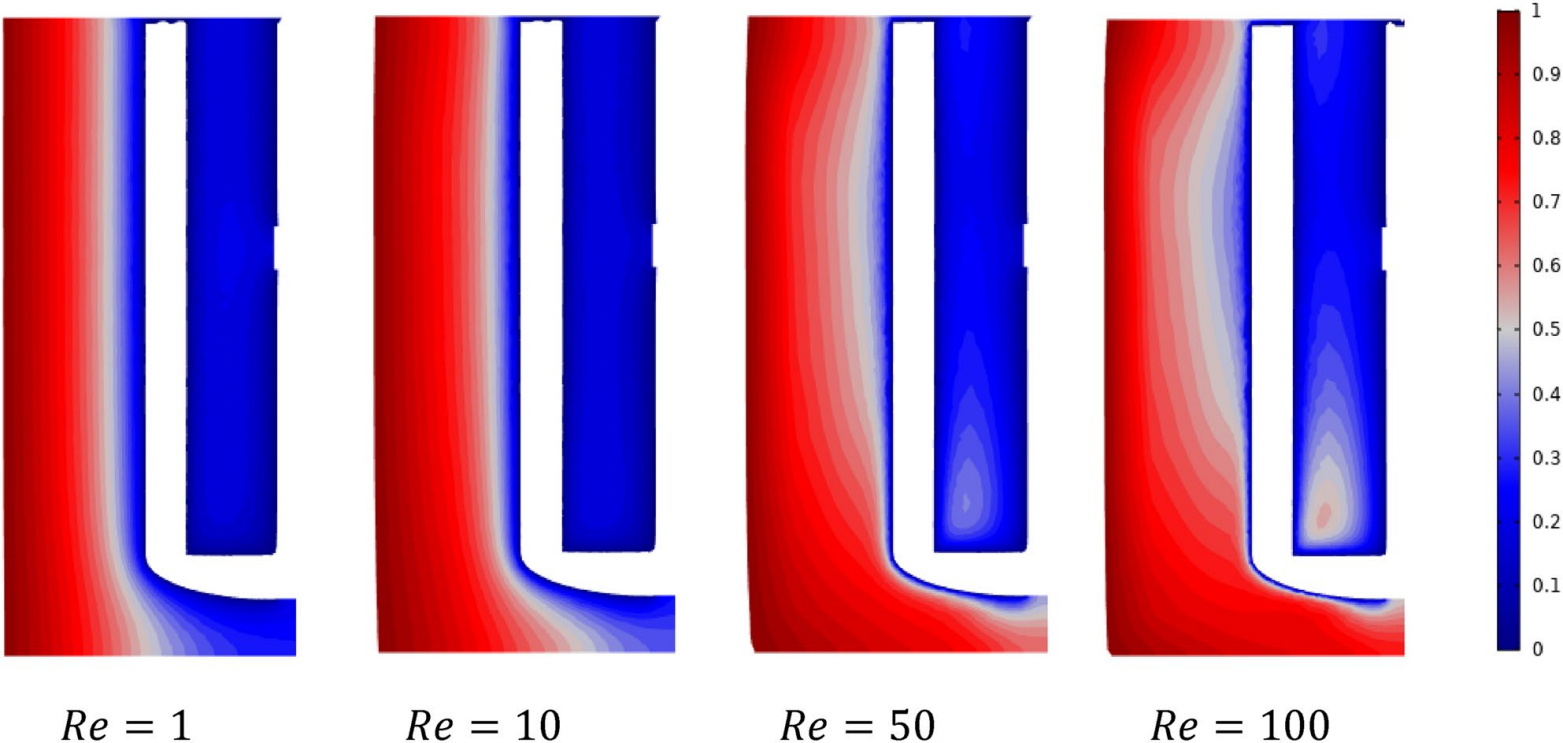
Contour velocity distribution in the horizontal impeller section on the stirred tank for different Reynolds numbers.

**Figure 13:**
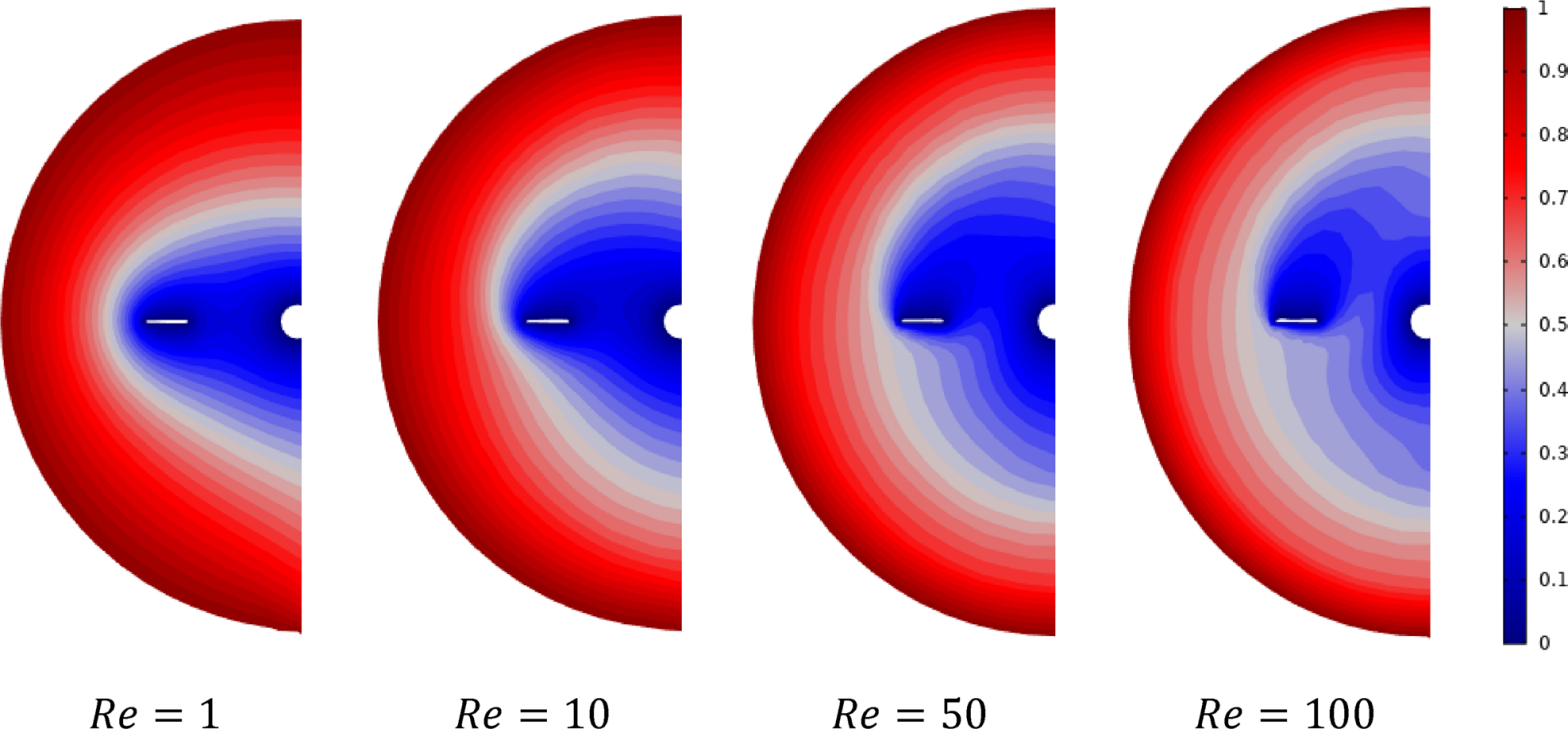
Contour velocity distribution in the horizontal impeller section on the stirred tank for different Reynolds numbers.

Figures 12 and 13 show the isotherm distribution in the stirred tank's vertical and horizontal section for different Reynolds values ($Re=1, 10, 50,$ and $100$). From the Figures, it can be seen at low inertia values $Re=1$ and $10$; the thermal flow is located between the tank's sidewall toward the blade impeller. By increasing the Reynolds number ($Re=50$ and $100$), an expansion of the thermal flow has been remarked; especially, a heated zone appears in the region between the blade impeller and shaft from the bottom side. In addition, a regularity in the path of the thermic difference domain (vertical direction) can be detected for the low Reynolds value $Re = 1$ and $10$. This thermic difference starts to change with the increase of inertia values ($50$ and $100$) from the axial to the radial direction, indicating a change in the thermal behavior inside the stirred tank.

The same remark can be seen in Fig. 13 at the horizontal view of the stirred vessel, regularity in the thermal gradient field direction for low Reynolds value $Re=1$ and $10$, and the thermal fields changing this direction, especially near the anchor region. Similar outcomes have been remarks in previous analyses where the thermic and hydrodynamical behaviors were investigated using 2-D numerical studies by Refs.^15,29–31^ and in the 3-D simulation probed by Pedrosa et al.^32^ and Gammoudi et al.^33^.

**Figure 14:**
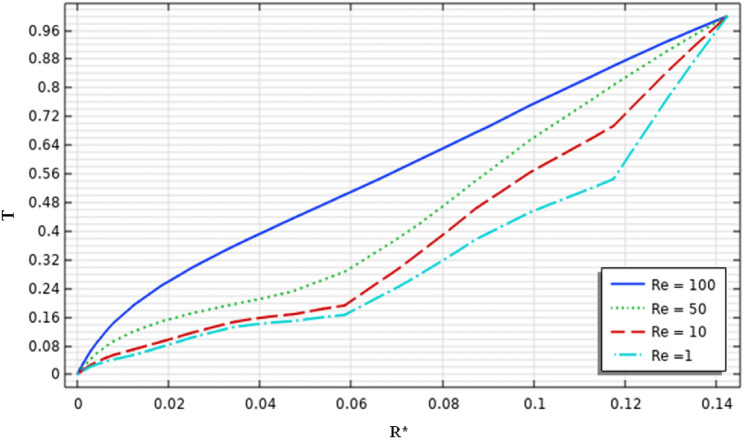
Temperature distribution on the vertical axis of the vessel.

**Figure 15:**
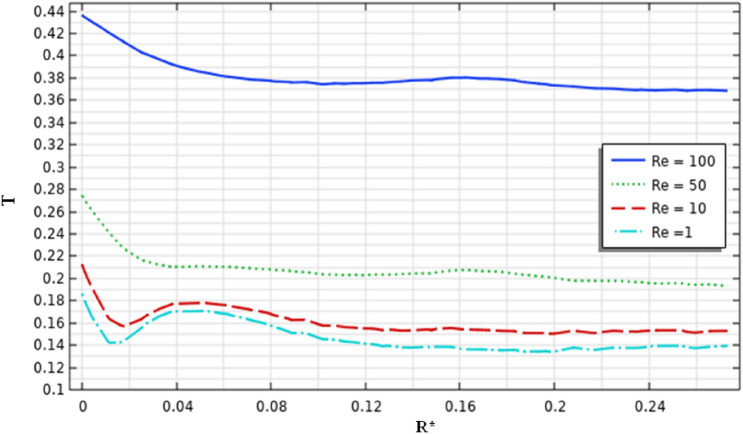
Temperature distribution on the vertical axis of the vessel.

**Figure 16:**
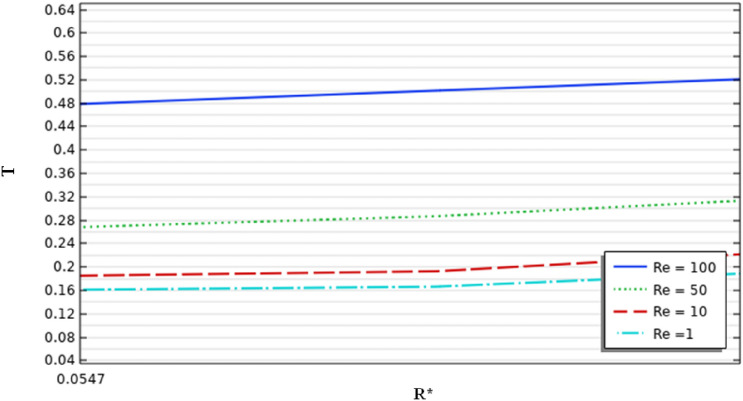
Temperature distribution on the vertical axis of the vessel.

Figures 14 and 15 illustrate the temperature variation along with the radial direction and the axial direction inside the stirred tank, respectively. From the result, it can be noted that the increase in the inertia value leads to an increase in the temperature value. From Fig. 16, by zooming the graph and verifying more precisely the temperature along the axial direction from the finding data ($Re=1$ the temperature up to $0.16$ and for $Re=100$ up to $0.48$ ) explains the increase of inertia multiplied by three times the thermal behavior acceleration inside the stirred vessel. A positive correlation was found between heat transfer and inertia variation along with the stirred tank. Interestingly, intensification progress on the thermal behavior was observed to explain the positive impact of inertia on enhancing thermal flow in the mixing system.

### Rheology effect

**Figure 17:**
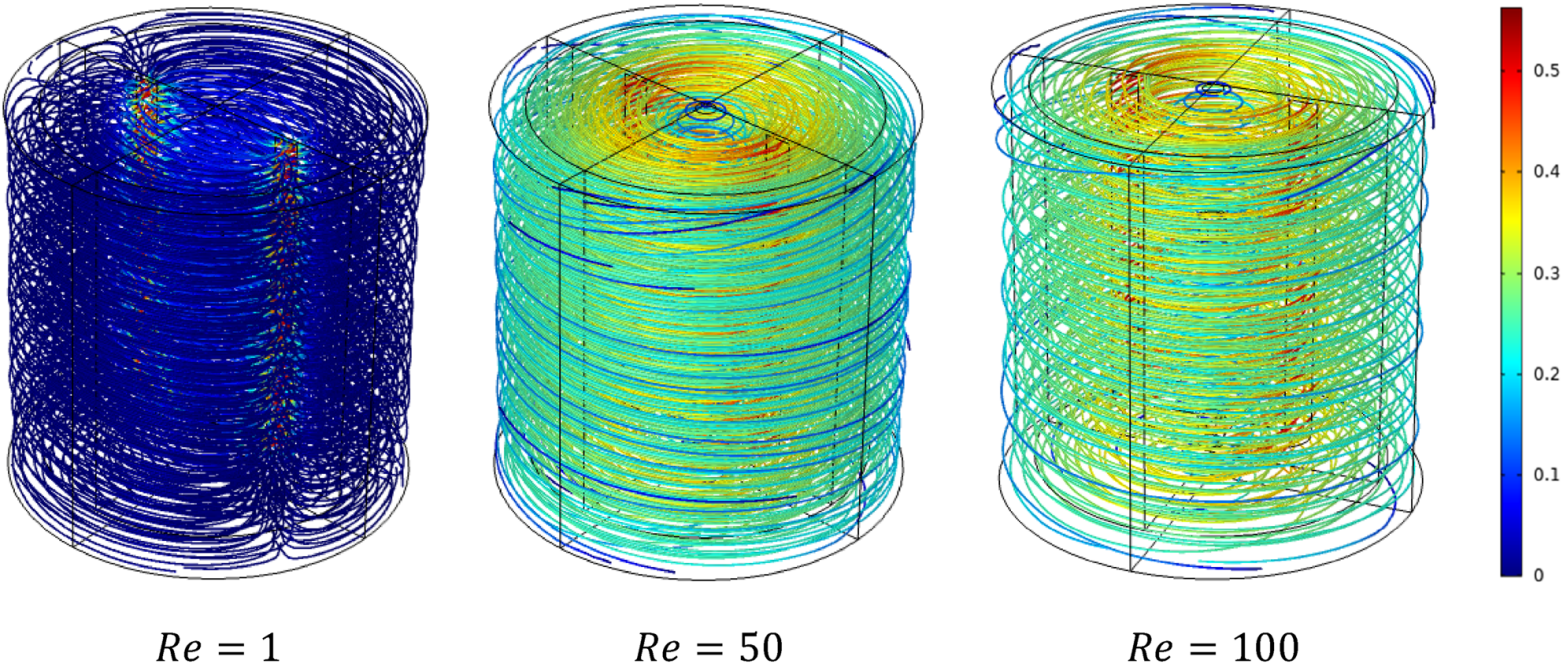
Streamline distribution along stirred vessel for $Bn=50$ with inertia variation value.

**Figure 18:**
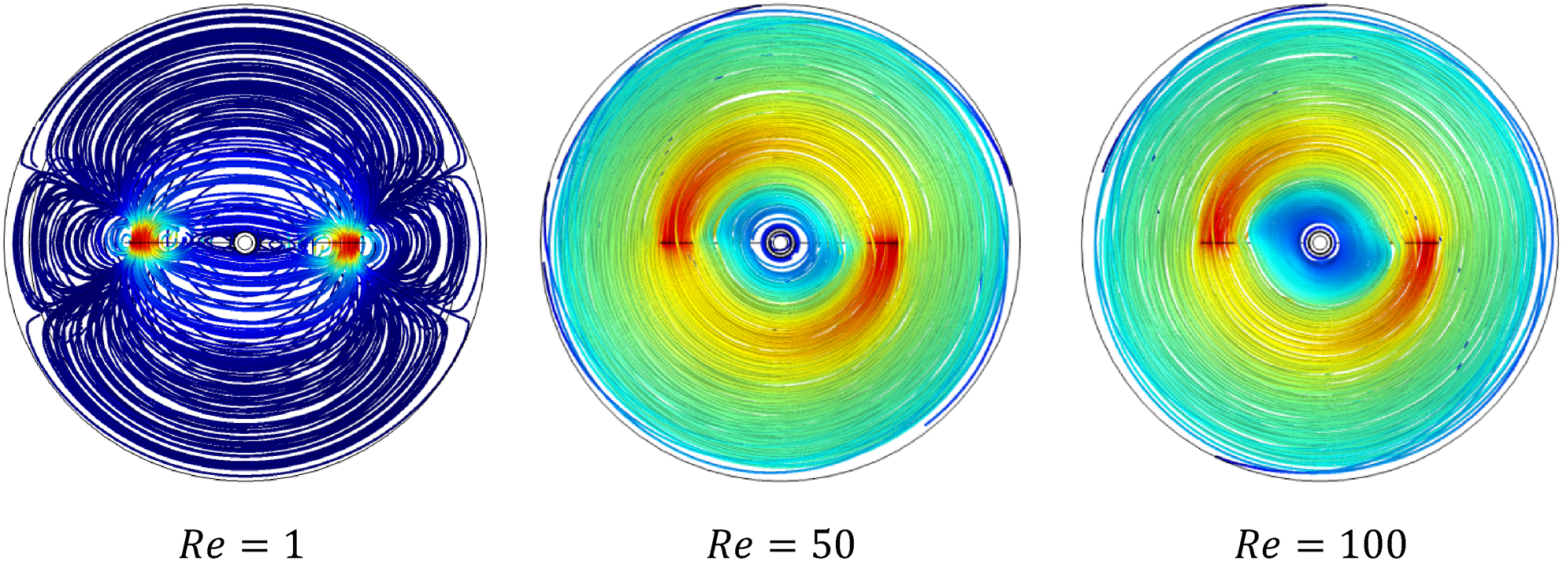
2D streamline distribution along stirred vessel for $Bn=50$ with inertia variation value.

**Figure 19:**
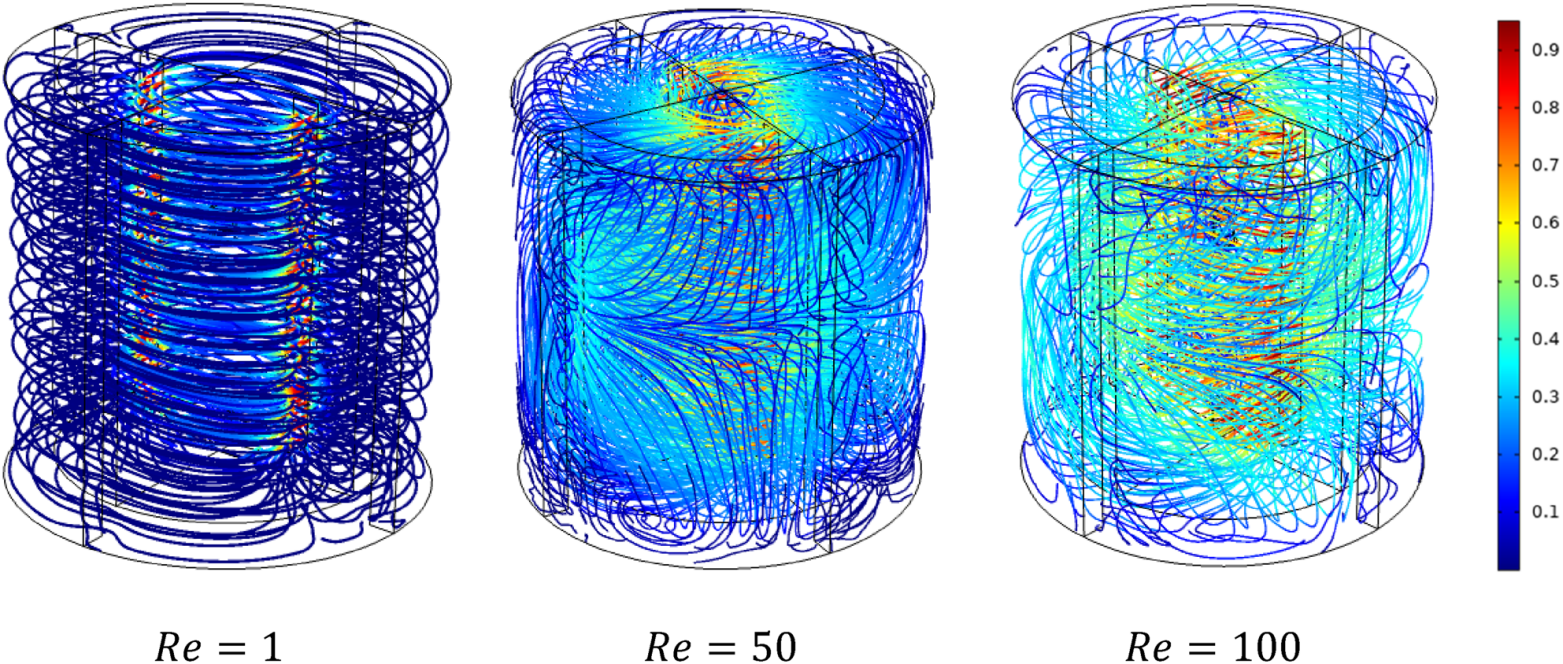
Streamline distribution along stirred vessel for $Bn=10$ with inertia variation values.

**Figure 20:**
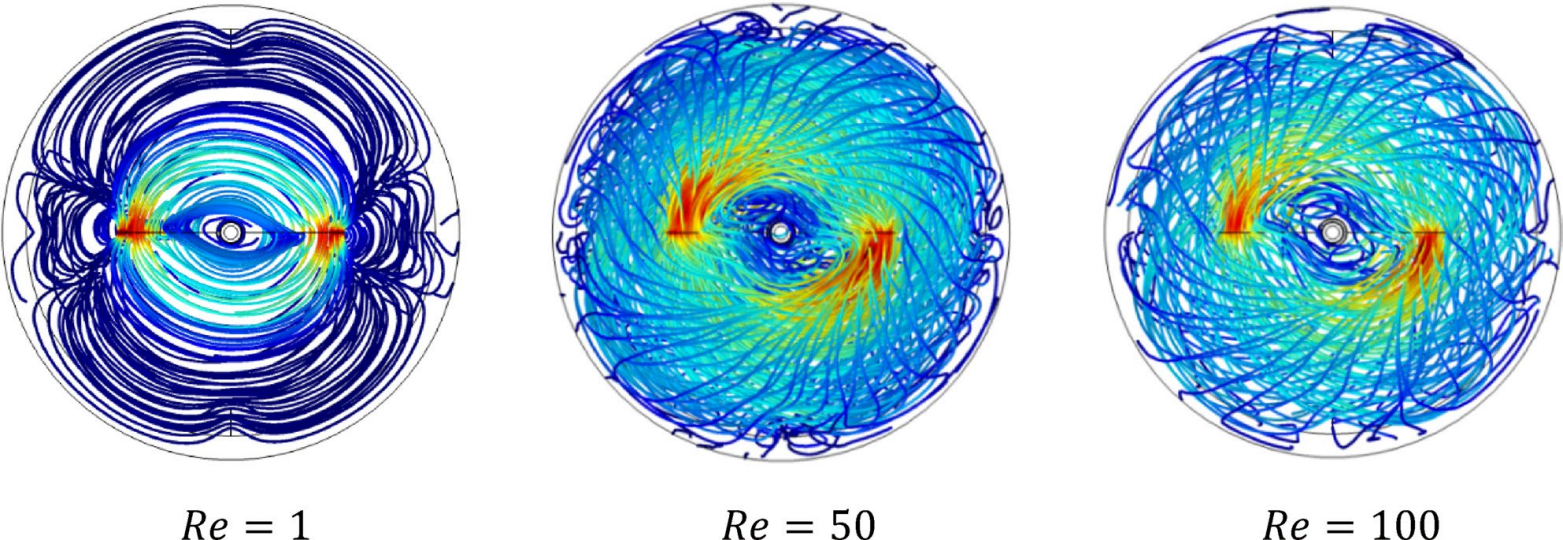
2D streamline distribution along stirred vessel for $Bn=10$ with inertia variation values.

**Figure 21:**
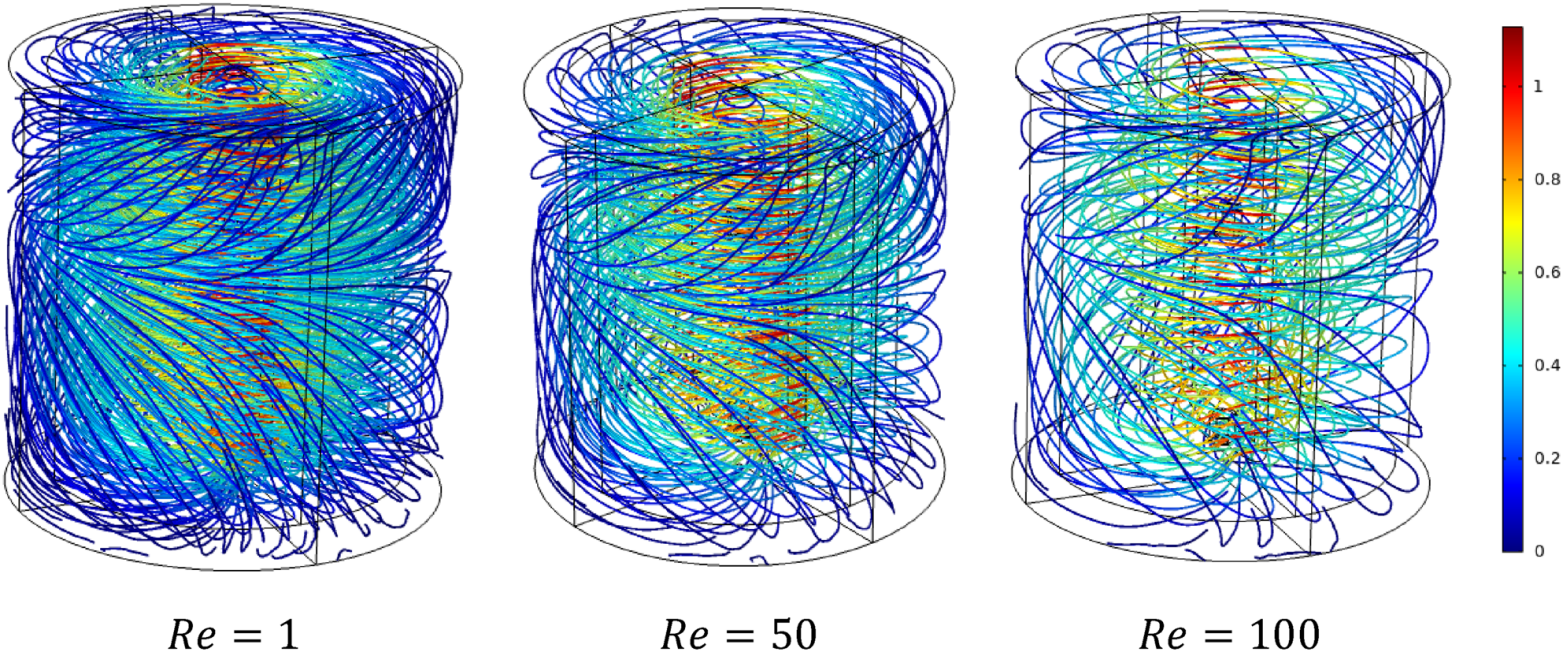
Streamline distribution along stirred vessel for $Bn=1$ with inertia variation values.

**Figure 22:**
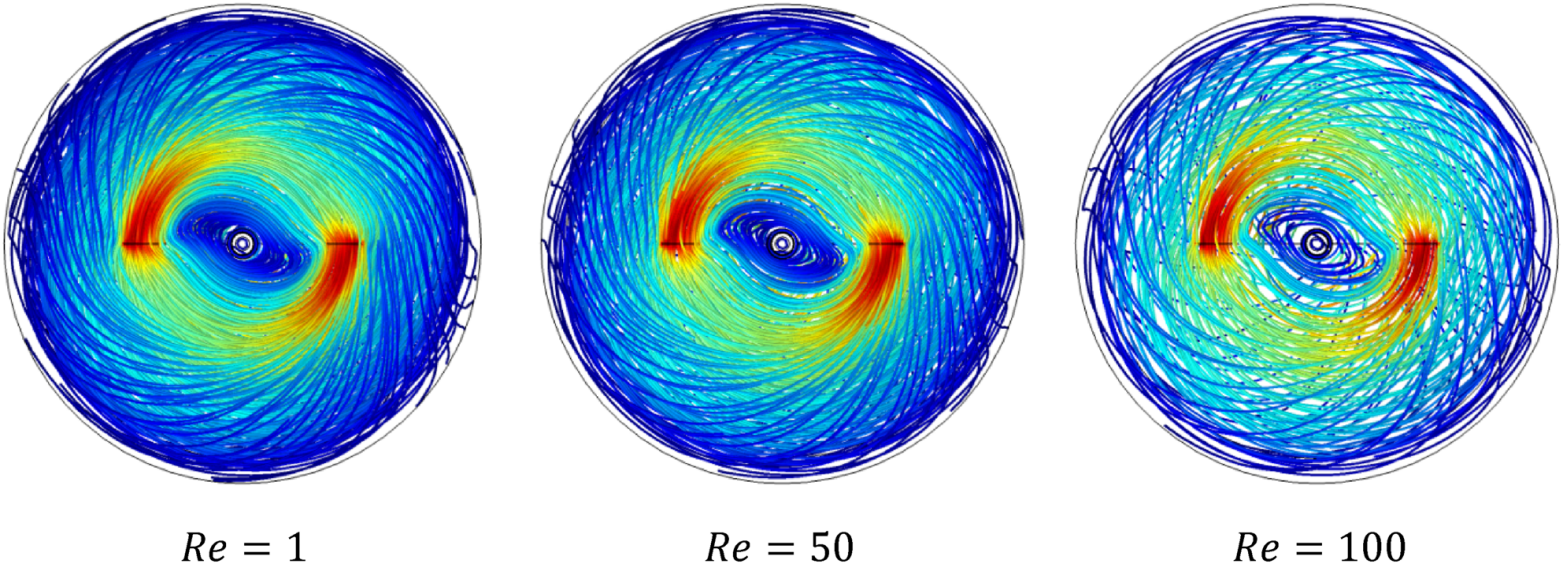
2D streamline distribution along stirred vessel for $Bn=1$ with inertia variation values.

Rheology is an important parameter affecting the thermo-hydrodynamic behavior within the agitated system; flow pattern, heat transfer, and power consumption are required inside a mixing system**.** In this case part, we analyze the effect of the Rheology parameter on the thermo-hydrodynamic structure by varying the plasticity value of this viscoplastic fluid. Figures 17, 18, 19, 20, 21, 22 illustrate the streamlined distribution throughout the stirred vessel for different plasticity values **(**$Bn=1, 10,$ and $50$**)**. As shown in Figs. 17 and 18, for a great plasticity value ($Bn=50$), the variation of the flow pattern is slowly along with the three cases of different inertia values. In the first case, a low inertia value ($Re =1$) vortex zone, as shown in the vicinity of the vessel, explains the existence of a stagnant region; in addition, the similarity of streamline distribution along all horizontal levels of the vessel affirms that the flow pattern is predominantly tangential in this case. The increase of inertia ($Re=50$ and $100$) eliminates this vortex zone and accelerates the flow field in a stirred tank. On the other hand, the flow pattern shape remains the same tangentially along the vessel despite the high inertia value.

An increase in flow intensity is remarked with the decrease in plasticity value ($Bn=10$) illustrated in Figs. 19 and 20. A radical change in the flow pattern in case inertia ($Re=50$, and $100$) was found in the streamlines' significant inclination toward the axial direction. For low plasticity value ($Bn=1$), it is clear to see no vortex was created near the blade region for ($Re=1$), and the flow quickly becomes axial with the increase of inertia value ($Re = 50$, and 100) as shown in Figs. 21 and 22. It can also be observed across the effect between inertia and plasticity. The decrease in plasticity leads to a change in flow pattern structure fast on the mixing system as a low inertia value.

It is apparent from these results in Figs. 17, 18, 19, 20, 21, 22 that there's a slow-moving inside stirred tank with a stagnant region in the vicinity of the vessel for high plasticity. The decrease in Bingham number leads the fluid to become efficient in shifting in a vertical path, which enhances the flowing inside the tank. In low Reynolds numbers, the existence of the vortex area nearby the shaft of the vessel and it's dissipated with the increase of Reynolds values; the same result obtained from Ameur^29^.

**Figure 23:**
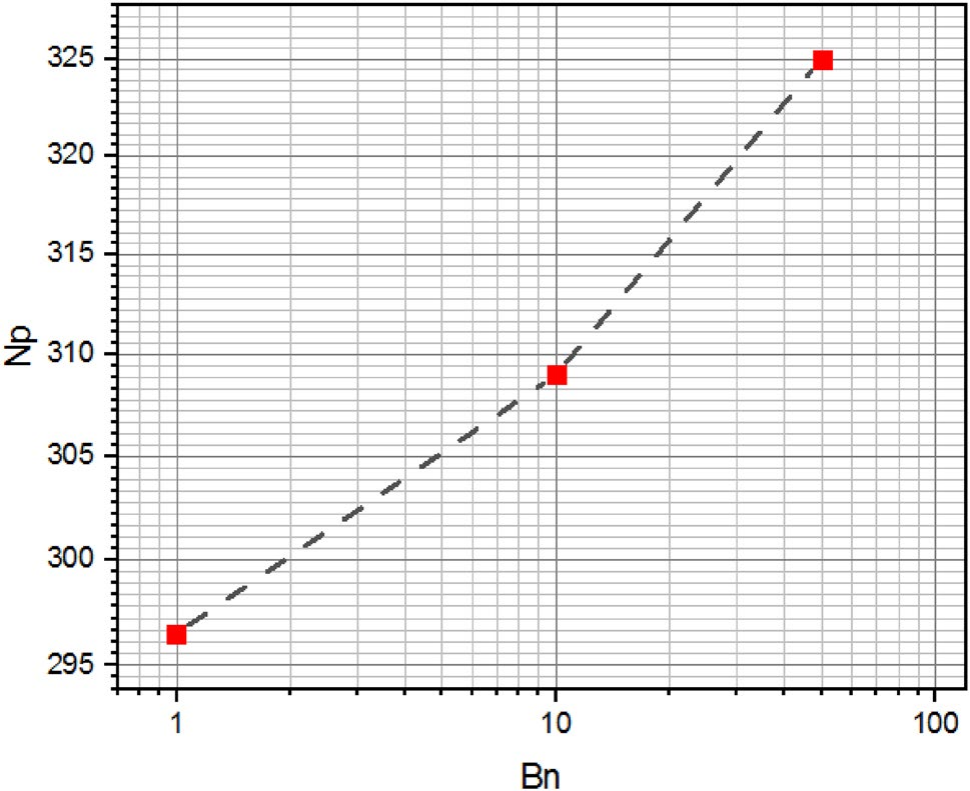
Power consumption versus Bingham number.

Figure 23 represents the power consumption as a function of plasticity parameters $Bn$. It can be seen that the increase in the plasticity value leads to an increase in the energy consumed in stirred system.

### Geometry design effect

In this study, the different geometrical design of anchor impellers has been introduced in the mixing system to analyze their impact on the thermal hydrodynamics comportment in a stirred tank. The anchor impeller classic was modified by adding an arm blade in the original shape at a different position. This work examined four different geometry combinations. CAI is for a classical anchor impeller; AI1 is for an anchor impeller with an additional blade in the center. AI2 illustrates the addition of two blades to the anchor impeller's center, while AI3 demonstrates the addition of three blades to the anchor impeller's center.

**Figure 24:**
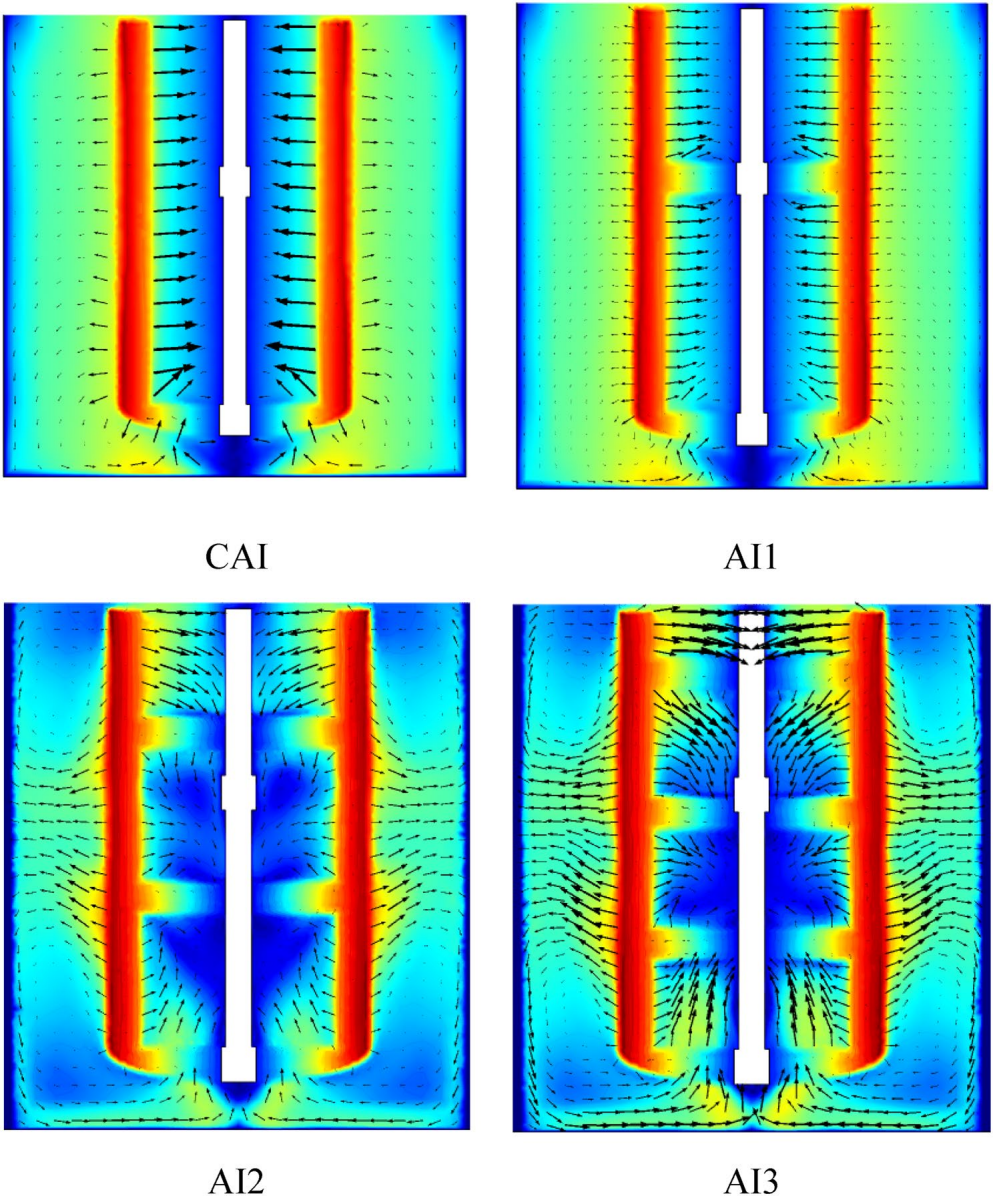
Velocity magnitude and vectors filed distribution on the vertical section of the vessel with different geometry configurations.

Figure 24 shows the velocity and vector field distribution on the vertical section in the vessel with different anchor impeller shapes, a significant increase in the well-moving zone with the rise of arm blade number. In CAI and AI1, the vectors flow filed are typically parallel for all levels of the stirred tank. This means a tangential flow found in most of the stirred systems for this case and the low-velocity value. With the increase of blade numbers (AI2 and AI3), a radical change in the flow pattern occurred with the appearance of axial flow. Axial flow resulted in pumping fluid from the bottom to the top of the stirred tank, resulting in an improvement in the flow pattern inside the stirred tank and a high vector field density along the vessel, which explains the existence of a large moving zone.

**Figure 25:**
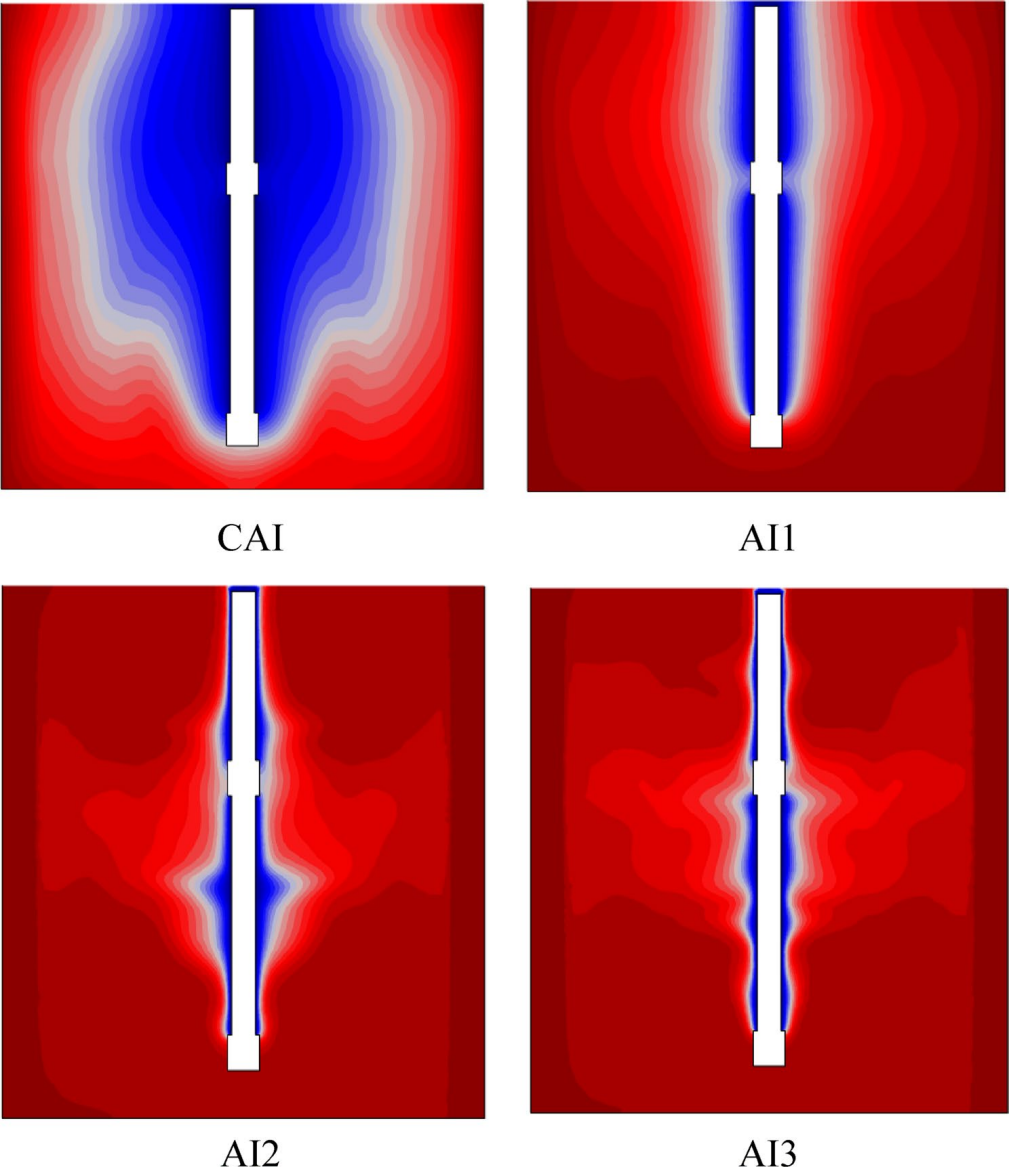
Isotherm distribution on the vertical section of the vessel with different geometry configurations.

Figure 25 demonstrates the outline of the isotherm in the vertical section of the tank for diverse geometry configurations. From the results obtained, it can be seen that the increase in blades number leads to an increased temperatures value along with the stirred tank. The isotherm pattern showed a deviation of the thermal gradient direction (irregular degradation in the thermal contour) in AI2 and AI3, compared with CAI and AI1, which have a regularity in the thermal gradient distribution. These are related to the effect of forced convection on the stirred tank and the impact of anchor impeller design. From Fig. 25, increasing the number of blades does not affect energy consumption. However, based on the previous findings, the impeller with three blades (AI3) may be selected as the most efficient because of the improvement in the heat transfer and acceleration flow field within the stirred tank.

**Figure 26:**
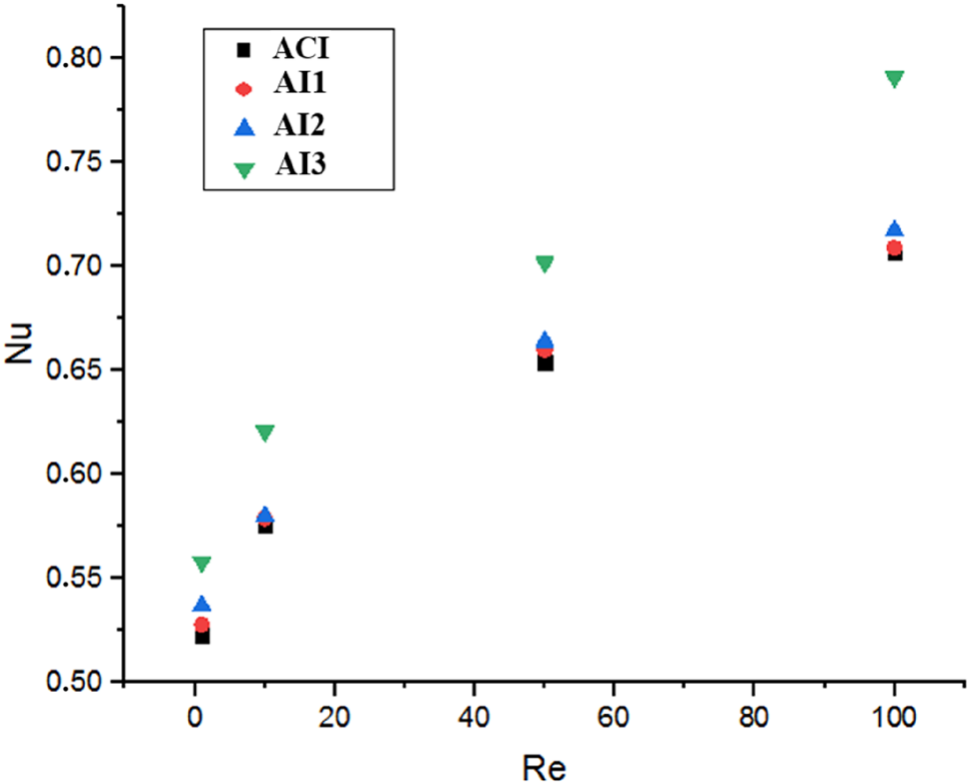
Nusselt number versus Reynolds number for different geometry configurations.

Figure 26 shows the Nusselt number variation as a function of the Reynolds number for different geometry cases. From the finding, the AI3 has a significant effect on the thermal behavior comparing with other cases, which also confirms the efficiency of this geometry configuration on the improvement and intensifies the thermal behavior inside the stirred tank. This case leads to an increase in the heat transfer flow with a rate of 10.46% for Reynolds number $Re=100$ as shown in Table 3.

**Table 3:** Nusselt number versus geometry configuration at $Re=100$.

	ACI	AI1	AI2	AI3
$Nu$	0.707	0.709	0.7173	0.781
$\Delta (Nu)\%$	–	0.28	1.4	10.46

## Conclusion

Numerical investigation of thermo-hydrodynamic behavior of viscoplastic (Casson–Papanastasiou model) stirring inside the cylindrical vessel by introducing new anchor impellers designed to improve the overall performances inside the stirred tanks. There were four different cases: CAI (classical anchor impeller), AI1 (anchor impeller shape with one added arm blade), AI2 (anchor impeller shape with two added arm blades), and AI3 (anchor impeller shape with three added arm blades).

This study aimed to demonstrate and analyze the effect of inertia and plasticity on the thermo-hydrodynamic structure (flow pattern, power consumption, and heat transfer) by varying the Reynolds number $Re$ from 1 to 100, Bingham number $Bn$ from1 to 50. besides the influence of geometry design in the overall stirred system parameters. The finding results revealed the following consequences:

- The flow pattern inside the stirred system changes proportionally with the inertia parameters, increasing the inertia value concomitant to an increase in the velocity inside the stirred tank.
- The flow is predominantly tangential with a low inertia value, especially for ($Re=1$); however, the rise in Reynolds value ($Re=50$ and $100$) leads to a change in the flow pattern from the tangential to the axial direction.
- The inertia variation has influenced heat transfer; a low temperature appears with low inertia values; moreover, with rising inertia $Re=200$, the heat ratio went up from 0.16 to 0.48, which means the inertia multiplied three times the heat transfer inside the stirred tank.
- While the height of the plasticity parameters causes a high energy cost in the stirred vessel, the inertia value increases, and the energy used within the vessel decreases. The power numbers value was remarkably near thanks to the anchor impeller's different geometrical shape, nevertheless.
- The finding results revealed that all geometry configurations have the same power energy consumption rate during the mixing operation, however the geometry configuration with three blades (AI3) significantly influences the improvement of flow pattern and enhances heat transfer by an increased rate of 10.46% over the other cases.

The current technique could be applied to a variety of physical and technical challenges in the future^34–40^.

## Data Availability

All data generated or analyzed during this study are included in this published article.

## List of symbols

$A$: Surface around the impeller (${\text{m}}^{2}$)
$Bn$: Bingham number (dimensionless)
$$D$$: Vessel diameter ($$\text{m}$$)
$Da$: Anchor impeller diameter ($\text{m}$)
$$Ds$$: Shaft diameter ($$\text{m}$$)
*F*_*x*_: *x*-Force direction (N)
*F*_*y*_: *y*-Force direction (N)
$$H$$: Tank height ($$\text{m}$$)
$\text{m}$: Stress growth parameter ($\text{s}$)
$N$: Rotational speed impeller ($\text{m}/\text{s}$)
$Np$: Power number (dimensionless)
$$Nu$$: Nusselt number
$$R$$: Vessel radius ($$\text{m}$$)
$Re$: Reynolds number (dimensionless)
$S$: Rate-of-deformation tensor
$$V{t}^{*}$$: Dimensionless velocity ($$V{t}^{*}=Vt/\pi ND$$)
$$T$$: Temperature ($$\text{K}$$)
${T}_{h}$: Hot wall temperature ($\text{K}$)
${T}_{c}$: Cold wall temperature ($\text{K}$)
$$w$$: Blade width ($$\text{m}$$)

## Greek symbols

$$\dot{\gamma }$$: Shear rate ($$1/\text{s}$$)
$\mu$: Dynamic viscosity (Pa s)
${\mu }_{0}$: Plastic viscosity (Pa s)
$$\Gamma$$: Torque ($$\text{Nm}$$)
$$\rho$$: Fluid density, ($$\text{kg}/\text{m}^{3}$$)
$\tau$: Shear stress (Pa)
$\Omega$: Impeller rotational speed (1/s)
